# EAGLE—A Scalable Query Processing Engine for Linked Sensor Data †

**DOI:** 10.3390/s19204362

**Published:** 2019-10-09

**Authors:** Hoan Nguyen Mau Quoc, Martin Serrano, Han Mau Nguyen, John G. Breslin, Danh Le-Phuoc

**Affiliations:** 1 Insight Centre for Data Analytics, National University of Ireland Galway, H91 TK33 Galway, Ireland; martin.serrano@insight-centre.org; 2 Information Technology Department, Hue University, Hue 530000, Vietnam; nmhan@hueuni.edu.vn; 3 Confirm Centre for Smart Manufacturing and Insight Centre for Data Analytics, National University of Ireland Galway, H91 TK33 Galway, Ireland; john.breslin@nuigalway.ie; 4 Open Distributed Systems, Technical University of Berlin, 10587 Berlin, Germany; danh.lephuoc@tu-berlin.de

**Keywords:** internet of things, graph of things, linked stream data, linked sensor data, semantic web, sensor network, spatial data, temporal RDF, RDF stores

## Abstract

Recently, many approaches have been proposed to manage sensor data using semantic web technologies for effective heterogeneous data integration. However, our empirical observations revealed that these solutions primarily focused on semantic relationships and unfortunately paid less attention to spatio–temporal correlations. Most semantic approaches do not have spatio–temporal support. Some of them have attempted to provide full spatio–temporal support, but have poor performance for complex spatio–temporal aggregate queries. In addition, while the volume of sensor data is rapidly growing, the challenge of querying and managing the massive volumes of data generated by sensing devices still remains unsolved. In this article, we introduce EAGLE, a spatio–temporal query engine for querying sensor data based on the linked data model. The ultimate goal of EAGLE is to provide an elastic and scalable system which allows fast searching and analysis with respect to the relationships of space, time and semantics in sensor data. We also extend SPARQL with a set of new query operators in order to support spatio–temporal computing in the linked sensor data context.

## 1. Introduction

The internet of things (IoT) is the network of physical objects embedded with sensors that are enabling real-time observations about the world as it happens. With estimates of there being 50 billion connected objects by 2020 [1], there will be an enormous amount of sensor observation data being continuously generated per second. These sensor observation data sources, in combination with existing data and services on the internet, are enabling a wide range of innovative and valuable applications and services in smart cities, smart grids, industry 4.0, intelligent transportation systems, etc. To be able to extract, meaningful information from heterogeneous sensor data sources in a variety of formats and protocols, the semantic web community has extended the Resource Description Framework (RDF) data model that has been widely used for representing web data, to connect dynamic data streams generated from IoT devices, e.g., sensor readings, with any relevant knowledge base, in order to create a single graph as an integrated database serving any analytical queries on a set of nodes/edges of the graph [2,3,4,5]. However, most current approaches using the RDF data model for managing sensor data, called linked sensor data, assume that RDF stores are able to handle queries on rapidly updating data streams in conjunction with massive volumes of data.

Data generated by sensors is also providing a meaningful spatio–temporal context, i.e., they are produced in specific locations at a specific time. Therefore, all sensor data items can be represented in three dimensions: the semantic, spatial and temporal dimensions. Consider the following example: “What was the average temperature during the past 30 min for Dublin city?”. This simple example poses an aggregate query across weather temperature readings from all weather stations in Dublin city. In this example, the semantic dimension describes the average temperature for Dublin city. The spatial dimension describes the place (Dublin city). The temporal dimension describes the time when the temperature values were generated (within the past 30 min). Unfortunately, supporting such multidimensional analytical queries on sensor data is still challenging in terms of complexity, performance, and scalability. In particular, these queries imply heavy aggregation on a large number of data points along with computation-intensive spatial and temporal filtering conditions. Moreover, the high update frequency and large volume natures of our targeted systems (around ten thousand updates per second on billions of records already in the store) will increase the burden of answering the query within some seconds or milliseconds. On top of that, by their nature, such systems need to scale to millions of sensor sources and years of data.

Motivated by such challenges, in this article, we present EAGLE, a scalable spatio–temporal query engine, which is able to index, filter, and aggregate a high throughput of sensor data together with a large volume of historical data stored in the engine. The engine is backed by distributed database management systems, i.e., OpenTSDB for temporal data and ElasticSearch for spatial data, and allows us to store a billion data points and ingest a large number of records per second while still being able to execute a spatio–temporal query in a timely manner. In summary, our contributions are as follows:

1. A proposed distributed spatio–temporal sub-graph partitioning solution which significantly improves spatio–temporal aggregate query performance.
2. An implementation of a comprehensive set of spatial, temporal and semantic query operators supporting computation of implicit spatial and temporal properties in RDF-based sensor data.
3. An extensive performance study of the implementation using large real-world sensor datasets along with a set of spatio–temporal benchmark queries.

The remainder of the article is organized as follows. In Section 2, we review related work on current solutions in existence. Section 3 describes the EAGLE engine architecture. The spatio–temporal storage model is given in Section 4. In Section 5, we present our spatio–temporal query language support through a series of examples. Section 6 elaborates on the implementation of our engine and its infrastructure to store and query sensor data. An experimental evaluation of this implementation follows in Section 7. Finally, we conclude and discuss future work in the last section.

## 2. Background and Related Work

### 2.1. Sensor Ontologies

During the last decade, an extensive amount of ontologies have been proposed, which aim to address the challenge of modeling a sensor network and its data, and also to tackle the heterogeneity problems associated with the hardware, software, and the data management aspect of sensors. More precisely, they provide a means to semantically describe the sensor networks, the sensing devices, the sensor data, and enable sensor data fusion.

The state-of-the-art approach in this area is the work from the Open Geospatial Consortium Sensor Web Enablement (OGC SWE) working group [6]. They have specified a number of standards that define formats for sensor data and metadata as well as sensor service interfaces. These standards allow the integration of sensor and sensor networks into the web, in what is called the sensor web. In particular, they provide a set of standard models and XML schema for metadata descriptions of sensors and sensor systems, namely the SensorML [7] and observations and measurements (O&M) models for data observed or measured by sensors [8,9]. A lack of semantic compatibility, however, is the primary barrier to realizing a progressive sensor web.

In [10], Amit et al. propose the semantic sensor web (SSW) that leverages current standardization efforts of the OGC SWE in conjunction with the semantic web activity of the World Wide Web Consortium W3C (www.w3.org/2001/sw/) to provide enhanced descriptions and meaning to sensor data. In comparison with the sensor web, the SSW addresses the lack of semantic compatibility by adding semantic annotations to the existing SWE standard sensor languages. In fact, these improvements aim to provide more meaningful descriptions to sensor data than SWE alone. Moreover, the SSW acts as a linking mechanism to bridge the gap between the primarily syntactic XML-based metadata standards of the SWE and the RDF/OWL-based metadata standards of the semantic web.

The work in [11] describes a practical approach for building a sensor ontology, namely OntoSensor, that uses the SensorML specification and extends the suggested upper merged ontology (SUMO) [12]. The objective of OntoSensor is to build a prototype sensor knowledge repository with advanced semantic inference capabilities to enable fusion processes using heterogeneous data. For that reason, in addition to reusing all SensorML’s concepts [7], OntoSensor provides additional concepts to describe the observation data, i.e., the geolocation of the observations, the accuracy of the observed data or the process to obtain the data.

Similar to OntoSensor, the W3C Semantic Sensor Network Incubator group (SSN-XG) has defined the SSN ontology [3] in order to overcome the missing semantic compatibility in OGC SWE standards, as well as the fragmentation of sensor ontologies into specific domains of application. The SSN ontology can be considered as a sort of standard for describing sensors and their resources with respect to the capabilities and properties of the sensors, measurement processes, observations, and deployment processes. It is worth mentioning that, although the SSN ontology provides most of the necessary details about different aspects of sensors and measurements, it does not describe domain concepts, time, location, etc. Instead, it can be easily associated with other sources of knowledge concerning, e.g., units of measurement, domain ontologies (agriculture, commercial products, environment, etc.). This helps to pave the way for the construction of any domain-specific sensors ontology. Because of its flexibility and adaptivity, the ontology has become more general and has been used in many research projects and applied to several different domains in recent years. Some of the most recently published works that utilize the SSN ontology are the OpenIoT Project [13], the FIESTA-IoT (http://fiesta-iot.eu/) [14], VITAL-IoT (http://www.vital-iot.eu/) and GeoSMA [15].

The broad success of the initial SSN led to a follow-up standardization process by the first joint working group of the OGC and the W3C. This collaboration aims to revise the SSN ontology based on the lessons learned over the past number of years and more specifically, to address changes in scope and audience, some shortcomings of the initial SSN, as well as technical developments and trends in relevant communities. The resulting ontology, namely the SOSA ontology [16], provides a more flexible coherent framework for representing the entities, relations, and activities involved in sensing, sampling, and actuation. The ontology is intended to be used as a lightweight, easy to use, and highly expendable vocabulary that appeals to a broad audience beyond the semantic web community, but that can be combined with other ontologies. The SOSA/SSN ontologies also form the core model that has been used to model our sensor data [17,18].

### 2.2. Triple Stores and Spatio–Temporal Support

The current standard query language for RDF, i.e., SPARQL 1.1, does not support spatio–temporal query patterns on sensor data. Recently, there have been several complimentary works towards supporting spatio–temporal queries on RDF. For example, to enable spatio–temporal analysis, in [19], Perry et al. propose the SPARQL-ST query language and introduce the formal syntax and semantics of their proposed language. SPARQL-ST is extended from the SPARQL language to support complex spatial and temporal queries on temporal RDF graphs containing spatial objects. With the same goal as SPARQL-ST, Koubarakis et al. propose st-SPARQL [20]. They introduce stRDF as a data model to model spatial and temporal information and the stSPARQL language to query against stRDF. Another example is [21], where Gutierrez et al. propose a framework that introduces temporal RDF graphs to support temporal reasoning on RDF data. In this approach, the temporal dimension is added to the RDF model. The temporal query language for temporal RDF graphs is also provided. However, the aforementioned works commonly focus on enabling spatio–temporal query features, but hardly any of them fully address the performance and scalability issues of querying billions of triples [22].

Regarding having to deal with the performance and scalability of RDF stores, many centralized and distributed RDF repositories have been implemented to support storing, indexing and querying RDF data, such as Clustered TDB [23], Inkling [24], RDFStore (http://rdfstore.sourceforge.net), Jena [25], and 4Store (http://4store.org). These RDF repositories are fast and able to scale up to many millions of triples or a few billion triples. However, none of the systems take the spatio–temporal features into consideration.

Toward supporting spatial queries on RDF stores, Brodt et al. [26] and Virtuoso (https://github.com/openlink/virtuoso-opensource) utilize RDF query engines and spatial indices to manage spatial RDF data. Reference [26] uses RDF-3x as the base index and adds a spatial index for filtering entities before or after RDF-3x join operations. Another example is OWLIM [27], which supports a geospatial index in its Standard Edition (SE). However, none of them systematically address the issue of elasticity and scalability for spatio–temporal analytic functions to deal with the massive volume of sensor data. The technical details and the index performance are also not mentioned in such system descriptions. Moreover, these approaches only support limited spatial functions, and the spatial entities have to follow the GeoRSS GML [28] model. Such systems are not aware of the temporal nature of linked sensor data that might be distributed over a long time span. For example, in our evaluations, most of the data is continuously archived for 10 months or even 10 years, for weather data. Therefore, such systems can easily run into scalability issues when the data grows. In one of our experiments [29], a triple store crashed after a few weeks ingesting weather sensor readings from 70,000 sensor stations and the system could not reliably answer any simple queries with a few billion triples in the store. Taking such limitations into consideration, the work presented in this article is a new evolution of our series of efforts [18,29,30,31,32,33] towards managing sensor data, together with other related work in the community. The main focus of this work is designing a query engine that is able to support complex spatio–temporal queries tailored towards managing linked sensor data, while the engine is also capable of dealing with the aforementioned performance and scalability issues. The design of such an engine is presented in the next section.

## 3. System Architecture

**Figure 1:**
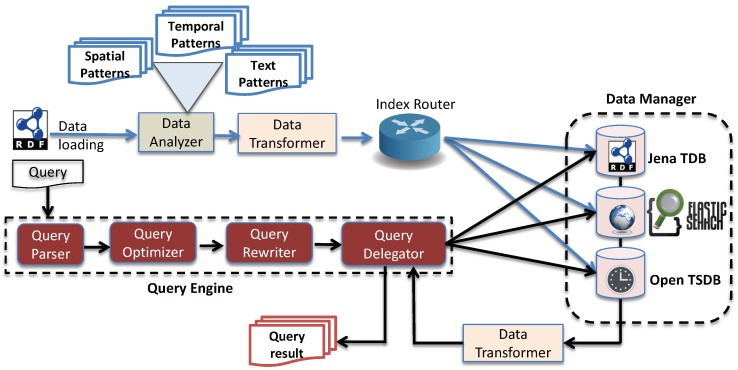
EAGLE’s architecture.

The architecture of EAGLE is illustrated in Figure 1. The engine accepts sensor data in RDF format as input and returns an output in SPARQL Result form (https://www.w3.org/TR/rdf-sparql-XMLres/). The general processing works as follows. When the linked sensor data is fed to the system, it is first analyzed by the data analyzer component. The data analyzer is responsible for analyzing and partitioning the input data based on the RDF patterns that imply the spatial and temporal context. The output sub-graphs of the data analyzer will be converted by the data transformer to the compatible formats of the underlying databases. The index router module then receives the transformed data and forwards them to the corresponding sub-database components in the data manager. In the data manager, we choose Apache Jena TDB (https://jena.apache.org/), OpenTSDB (http://opentsdb.net/) [34], and ElasticSearch (https://www.elastic.co/) as underlying stores for such partitioned sub-graphs.

To execute the spatio–temporal queries, a query engine module is introduced. The query engine consists of several sub-components that are responsible for parsing the query, generating the query execution plan, rewriting the query into sub-queries and delegating sub-query execution processes to the underlying databases. The data manager executes these sub-queries and returns the query results. After that, the data transformer transforms the query results accordingly to the format that the query delegator requires. Details of EAGLE’s components are described in the following subsections.

### 3.1. Data Analyzer

**Figure 2:**
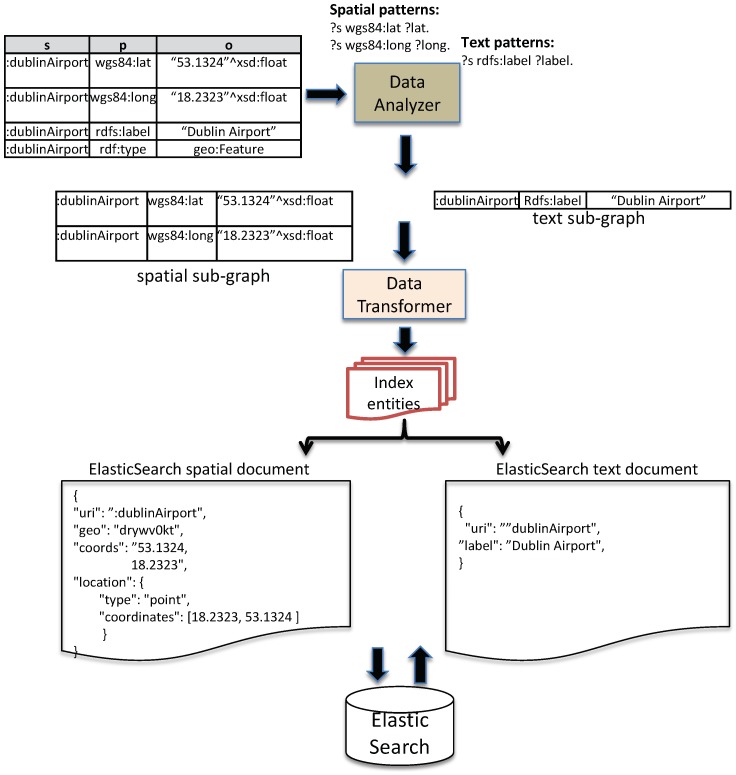
Transform spatial and text sub-graphs to ElasticSearch documents.

As mentioned above, for the input sensor data in RDF format, the data analyzer evaluates and partitions them to the corresponding sub-graphs based on their (spatial, temporal or text). Data characteristics are specified via a set of defined RDF triple patterns. In EAGLE, these RDF triple patterns are categorized into three types: spatial patterns, temporal patterns, and text patterns. The spatial patterns are used to extract the spatial data that need to be indexed. Similarly, temporal patterns extract the sensor observation value along with its timestamp. The text patterns extract the string literals. An example of the partitioning process is illustrated in Figure 2. In this example, we define (?s wgs84:lat ?lat. ?s wgs84:long ?long) and (?s rdfs:label ?label) as the triple patterns used for extracting spatial and text data, respectively. For instance, assume that the system receives a set of input triples shown in Listing 1.


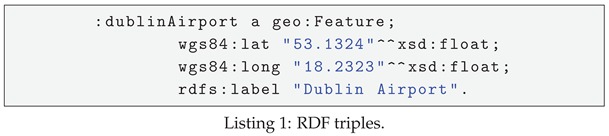


As demonstrated in Figure 2, the two triples (:dublinAirpot wgs84:lat “53.1324”⌃⌃xsd:float.:dublinAirpot wgs84:long “18.2323”⌃⌃xsd:float) are found to match the defined spatial patterns (?s wgs84:lat ?lat. ?s wgs84:long ?long), and thus are extracted as a spatial graph. Similarly, we have the text sub-graph (:dublinAirport rdfs:label ”Dublin Ariport”) extracted. These sub-graphs will be transformed into compatible formats to be used by the indexing process in the data manager. The data transformation process will be presented in the following section.

### 3.2. Data Transformer

The Data Transformer is responsible for converting the input sub-graphs received from the data analyzer to the index entities. The index entities are the data records (or documents) constructed to a compatible data structure so that they can be indexed and stored in the data manager. Returning to the example in Figure 2, the data transformer transforms the spatial sub-graph and text sub-graph into ElasticSearch documents. In addition to transforming the sub-graphs into the index entities, the data transformer also has to transform the query outputs generated by the data manager to the format that the query delegator requires.

### 3.3. Index Router

The index router receives the index entities generated by the data transformer and forwards them to the corresponding database in the data manager. For example, the spatial and text index entities will be routed to ElasticSearch to index and ones that have temporal values will be transferred to the OpenTSDB cluster. For the index entities that do not match any spatial or temporal patterns, they will be stored in the normal triple store. Due to the fact that access methods can vary across different databases, the index router, therefore, has to support multiple access protocols such as Rest APIs, JDBC, MQTT, etc.

### 3.4. Data Manager

Rather than rebuilding the spatio–temporal indices and functions into one specific system, our data manager module adopts a loosely coupled hybrid architecture that consists of different databases for managing different partitioned sub-graphs. More precisely, we used ElasticSearch to index the spatial objects and text values that occur in sensor metadata. Similarly, we used a time-series database, namely OpenTSDB, for storing temporal observation values. The reasons for choosing ElasticSearch and OpenTSDB can be explained as follows: (1) ElasticSearch and OpenTSDB both provide flexible data structures which enable us to store sub-graphs which share similar characteristics but have different graph shapes. For example, stationA and stationB are both spatial objects but they have different spatial attributes (i.e., point vs. polygon, names vs. label, etc.). Moreover, such structures also allow us to dynamically add a flexible number of attributes in a table without using list, set, or bag attributes or redefining the data schema. (2) ElasticSearch supports spatial and full-text search queries. Meanwhile, OpenTSDB provides a set of efficient temporal analytical functions on time-series data. All of these features are the key-point requirements for managing sensor data. (3) Finally, these databases offer clustering features so that we are able to address the “big-data” issue, which is problematic for traditional solutions when dealing with sensor data.

For the non-spatio–temporal information that does not need to be indexed in the above databases, this will be stored in the native triple store. We currently use Apache Jena TDB to store such generic data. In the case of a small size dataset, it can be easily loaded into the RAM of a standalone workstation for the sake of boosting performance.

#### 3.4.1. Spatial-Driven Indexing

To enable querying of spatial data, we transform the sub-graph that contains spatial objects as a geo document and store it in ElasticSearch. Figure 2 demonstrates a process that transforms a semantic spatial sub-graph to an ElasticSearch geo document. Please be aware that, along with spatial attributes, ElasticSearch also allows the user to add additional attributes such as date-time, text description, etc. This advanced feature allows us to develop a more complex filter that can combine spatial filters and full-text search in a query.

The ElasticSearch geo document structure is shown in Listing 2. In this data structure, location is an ElasticSearch spatial entity used to describe geo–spatial information. It has two properties: type and coordinates. Type can be point, line, polygon, envelope while coordinates can be one or more arrays of longitude/latitude pair. Details of the spatial index implementation will be discussed in Section 5.1.


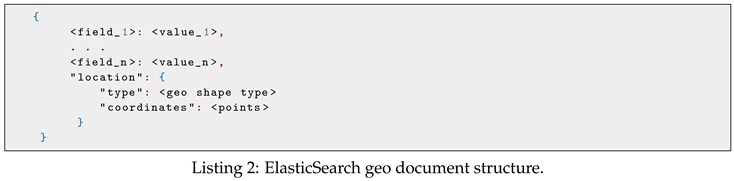


#### 3.4.2. Temporal-Driven Indexing

A large amount of sensor observation data is fed as a time-series of numeric values such as temperature, humidity and wind speed. For these time-series data, we choose OpenTSDB (Open Time-Series Database) as the underlining scalable temporal database. OpenTSDB is built on top of HBase [35] so that it can ingest millions of time-series data points per second. As shown in Figure 1, input triples which are comprised of numeric values and time-stamps are analyzed and extracted based on the predefined temporal patterns. Based on this extracted data, an OpenTSDB record is constructed and then stored in OpenTSDB tables.

In addition to the numeric values and timestamps, additional information can be added to each data record of OpenTSDB. Such information also can be used to filter the temporal data. Additional information is selected by their regular use for filtering data in SPARQL queries. For example, a user might want to filter data by type of sensor, type of reading, etc. The data organization and schema design in OpenTSDB will be discussed in Section 4.

### 3.5. Query Engine

As shown in the EAGLE architecture in Figure 1, the query processing of EAGLE is performed by the query engine that consists of a query parser, a query optimizer, a query rewriter and a query delegator. It is important to mention that our query engine is developed on top of Apache Jena ARQ. Therefore, the query parser is identical to the one in Jena. The query optimizer, query rewriter and query delegator have been implemented by modifying the corresponding components of Jena. For the query optimizer, in addition to Apache Jena’s optimization techniques, we also propose a learning optimization approach that is able to efficiently predict a query execution plan for an unforeseen given spatio–temporal query. Details of our approach can be found in our recent publication [33].

The query engine works as follows. First, for a given query, the query parser translates it and generates an abstract syntax tree. Note that, we have modified the query parser so that it can adapt our spatio–temporal query language. Next, the syntax tree is then mapped to the SPARQL algebra expression, resulting in a query tree. In the query tree, there are two types of nodes, namely non-leaf nodes and leaf nodes. The non-leaf nodes are algebraic operators such as joins, and leaf nodes are the variables present in the triple patterns of the given query. Following the SPARQL Syntax Expressions (https://jena.apache.org/documentation/notes/sse.html), Listing 3 presents a textual representation of the query tree corresponding to the spatio–temporal query in Example 5 of Section 6.


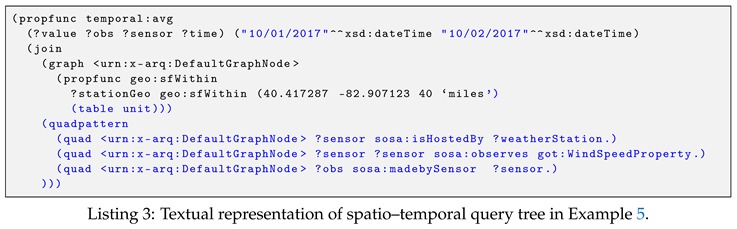


Please be aware that the query tree generated by the query parser is just a plain translation of the initial query to the SPARQL algebra. At this stage, there is no optimization technique being applied yet. After that, the query tree is processed by the query optimizer. This component is responsible for determining the most efficient execution plan with regard to the query execution time and resource consumption. After having a proper execution plan, it is passed to the query rewriter for any further processing needed. Basically, the query rewriter rewrites the query operators to the compatible query language of the underlying database. In the next step, the query delegator delegates these rewritten sub-queries to the corresponding database in the data manager. For example, the sub-query that contains the spatial operator or full-text search will be evaluated by ElasticSearch, while the temporal operator is executed by OpenTSDB. For the non-spatio–temporal queries, they are processed by Jena. After having the sub-queries executed, the query results need to be transformed to the format that the query delegator requires. The query delegator then performs any post-processing actions needed. The final step involves formatting the results to be returned to the user.

## 4. A Spatio–Temporal Storage Model for Efficiently Querying on Sensor Observation Data

As mentioned in Section 3, we chose OpenTSDB as an underlying temporal database for managing sensor observation data. In this section, we present a preliminary design of the OpenTSDB data schema used for storing these data sources. Due to the data-centric nature of wide column key-value stores of OpenTSDB, there are two most important decisions on storage model design that can affect to the system performance, which are: the form of the row keys and the partition of data. This section will present, in detail, our decisions for rowkey design and the data partitioning strategy that aim to enhance the data loading and query performance on sensor observation data.

### 4.1. Opentsdb Storage Model Overview

**Figure 3:**
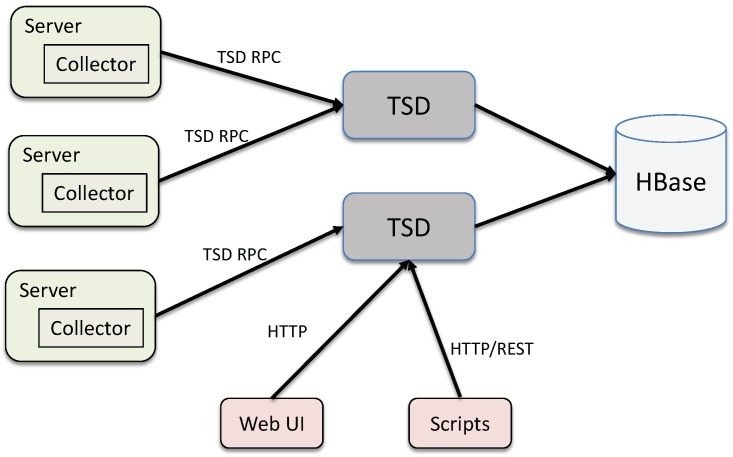
OpenTSDB architecture.

OpenTSDB is a distributed, scalable, time-series database built on top of Apache HBase [35], which is modeled after Google’s BigTable [36]. It consists of a time-series daemon (TSD) along with a set of command-line utilities. Data reading and writing operations in OpenTSDB are primarily achieved by running one or more of the TSDs. Each TSD is independent. There is no master, no shared state so that many TSDs can be deployed at the same time, depending on the loading throughput requirement. The OpenTSDB architecture is illustrated in Figure 3.

**Figure 4:**
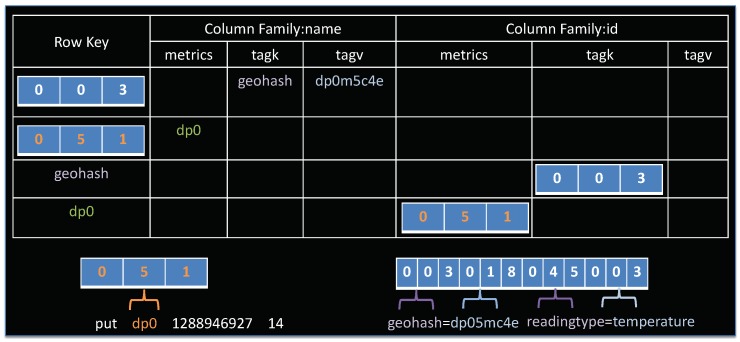
OpenTSDB *tsdb-uid* table.

Data in OpenTSDB are stored in a HBase table. A table contains rows and columns, much like a traditional database. A cell in table is a basic storage unit, which is defined as <RowKey,ColumnFamily:ColumnName,TimeStamp>. There are two tables in OpenTSDB, namely tsdb and tsdb-uid. The tsdb-uid table is used to maintains an index of globally unique identifiers (UID) and values of all metrics and tags for data points collected by OpenTSDB. In this table, two columns exist, one called “name” that maps an UID to a string, and another table, denoted as “id”, mapping strings to UIDs. Each row in the column family will have at least one of following three columns with mapping values: metrics for mapping metric names to UIDs, tagk for mapping tag names to UIDs, tagv for mapping tag values to UIDs. Figure 4 illustrates the logical view of tsdb-uid table.

**Figure 5:**
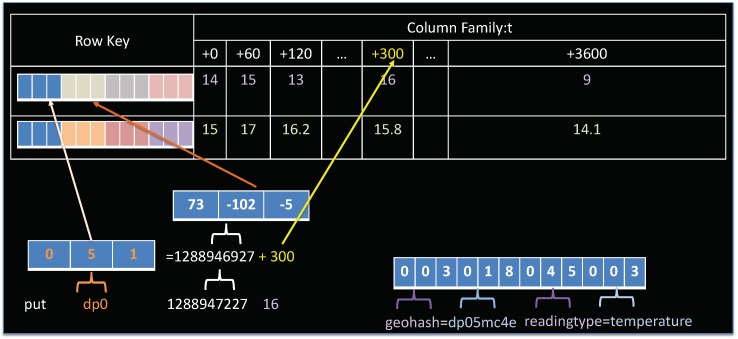
OpenTSDB tsdb table.

A central component of OpenTSDB architecture is the tsdb table that stores our time-series observation data. This table is originally designed to not only support time-based queries but also to allow additional filtering on metadata, represented by *tag* and *tag value*. This is accomplished through careful design of the rowkey. As described in Table 1, an OpenTSDB rowkey consists of three bytes for the metric id, four bytes for the base timestamp, and three bytes each for the tag name ID and tag value ID, repeated. Figure 5 presents an example of tsdb rowkey. As shown in this figure, the schema contains only a single column family, namely “t”. This is due to the requirement of HBase that a table has to contain at least one column family [37]. In OpenTSDB, the column family is not so important as it does not affect the organization of data. The column family “t” might consist of one or many column qualifiers representing delta elapse from the base timestamp. In this example, 16 is the value, *1288946927* is the base timestamp and column qualifier *+300* is the delta elapse from base timestamp.

### 4.2. Designing a Spatio–Temporal Rowkey

In order to make well-informed choices of the rowkey design, we first identified common data access patterns required by the user application when querying the sensor observation data. In the following, we enumerated a few common queries that can be expected by the realistic sensor-based applications presented in [4,13,38,39]:

- A user may request meteorological information of an area over a specific time interval. The query may include more than one measurement values, i.e., humidity, wind speed along with the temperature.
- A user may request the average observation value over a specific time interval using variable temporal granularity i.e., hourly, daily, monthly, etc.
- A user may request statistical information about the observation data that are generated by a specific sensor station.
- A user may ask for statistical information, such as the hottest month over the last year for a specific place of residence. Such queries can become more complex if the residence address is not determined by city name or postal code but by its coordinate.

There can be different rowkey design approaches for answering the aforementioned queries. Nevertheless, to have fast access to a relevant data based on the rowkey, there are two points needed to be taken into consideration when designing a rowkey schema for storing sensor observation data in OpenTSDB table: (1) data should be evenly distributed across all RegionServers to avoid the region hot-spotting performance [37]. Note that, a bad key design will lead to sub-optimal load distribution. The solution to address this issue will be presented in Section 4.3. (2) The spatio–temporal locality of data should be preserved. In other words, data of all the sensor that locate within the same area should be stored in the same partitions on the disk. The latter is essential in order to accelerate range scans since users will probably request data of a specific area over a time interval instead of just a single point in time.

Starting with the row key schema, we have to decide what information and in which order will be stored in the row key. Since spatial information is usually the most important aspect of user queries, encoding the sensor location in rowkey is prioritized. In this regard, a geohash algorithm is selected. Recall that a geohash is a function that turns the latitude and longitude into a hash string. A special feature of geohash is that, for a given geohash prefix, all the points within the same space match the common prefix. To make use of this feature, we encode the first three characters of geohash prefix as the metric uid of our rowkey schema. The length of the geohash prefix that is used to encode the metric uid can be various, depending on the data density. Data stored in the tsdb table are sorted on rowkey, thus, encoding geohash as metric uid, which is the first element of rowkey, ensures the data of sensor stations close to each other in space are close to each other on disk. Next, we append the measurement timestamp as the second element of a rowkey in order to preserve temporal ordering. At this stage, we accomplish the goal (2).

**Figure 6:**
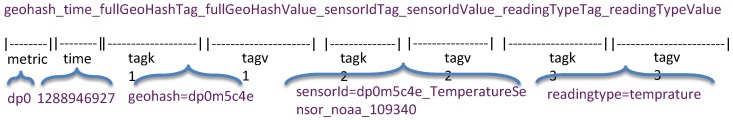
OpenTSDB rowkey design for storing observation data.

After defining the first two elements of the row key, the tag names and tag values must be specified. In OpenTSDB, tags are used for filtering data. Based on the summary of common data access patterns above, we recognize that users may filter data by either a detailed location, or by a specific sensor, or by a single type of sensor reading. Therefore, the following tags are defined: (1) the geohash tag to store the full geohash string representing sensor station location; (2) the sensorId to present the full IRI (Information Resource Identifier) of sensor that generates corresponding observation data; (3) the readingtype to indicate the observed property of observation data. These tags are then concatenated after the rowkey. The full form of our proposed row key design is depicted in Figure 6.

### 4.3. Spatio–Temporal Data Partitioning Strategy

Data partitioning has a significant impact on parallel processing platforms like OpenTSDB. If the sizes of the partitions, i.e., the amount of data per partition, are not balanced, a single worker node has to perform all the work while other nodes idle. To avoid this imbalance performance, in this section, we will present our data partitioning strategy that split data into multiple partitions and also exploits the spatio–temporal characteristics of sensor data.

**Figure 7:**
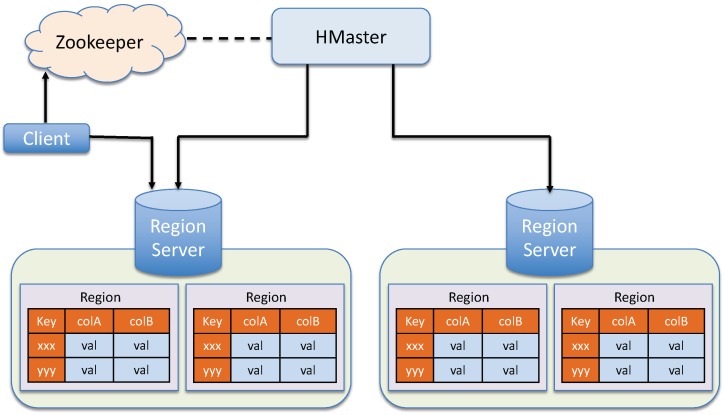
HBase tables.

As mentioned earlier, we store observation data in OpenTSDB *tsdb* table, which is originally an HBase table. By design, an HBase table can consist of many regions. A region is a table storage unit, that contains all the rows between the start key and the end key assigned to that region. Regions are managed by the Region Servers, as illustrated in Figure 7. Note that, in HBase, each region server serves a set of regions, and a region can be served only by a single region server. The HMaster is responsible to assign regions to region servers in the cluster.

**Figure 8:**
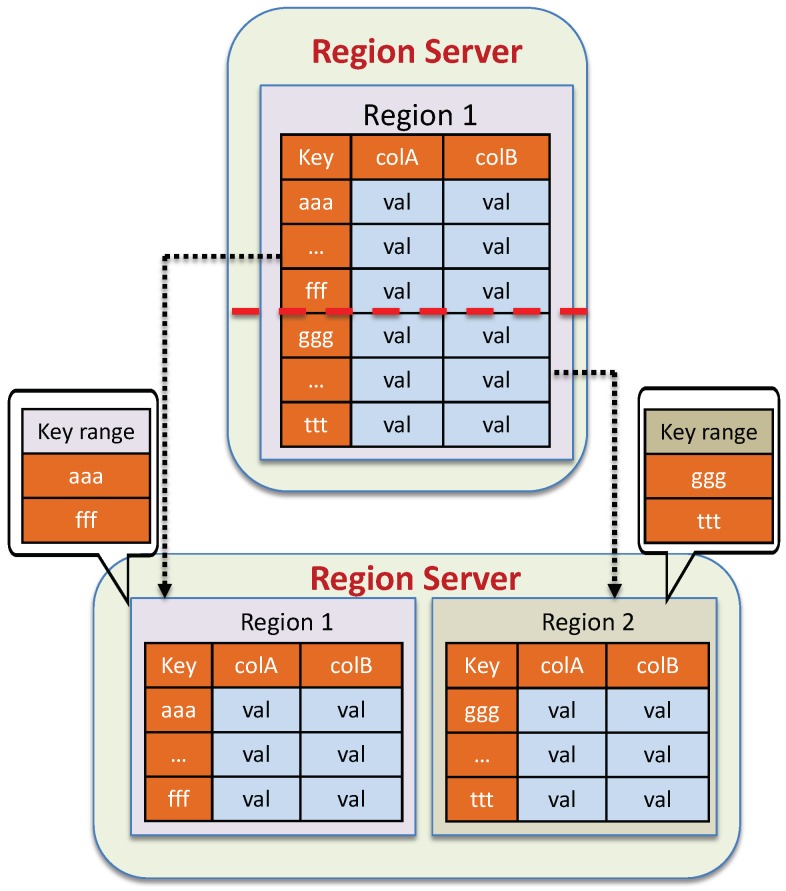
HBase table splitting.

Initially, when a table is created, it is allocated with a single region. Data are then inserted into this region. If the number of data records stored in this region exceeds the given threshold, HBase will partition it into two roughly equal-sized child regions. As more and more data are inserted, this splitting operation is performed recursively. Figure 8 describes the table splitting in HBase.

Basically, table splitting can be performed automatically by HBase. The goal of this operation is to avoid hot-spotting performance. However, table splitting is a costly task and can result in latency increased, especially during heavy write loads. In fact, splitting is typically followed by regions moving around to balance the cluster, which adds to the overhead and heavily affects to cluster performance. Therefore, to avoid this costly operation, we partition the tsdb table at the time of table creation using the pre-splitting method. For different data sources, the data partitioning strategy might be varied, as it is very dependent upon the rowkey distribution. Therefore, a good rowkey design is also a key factor in the effectiveness of a partitioning strategy.

**Figure 9:**
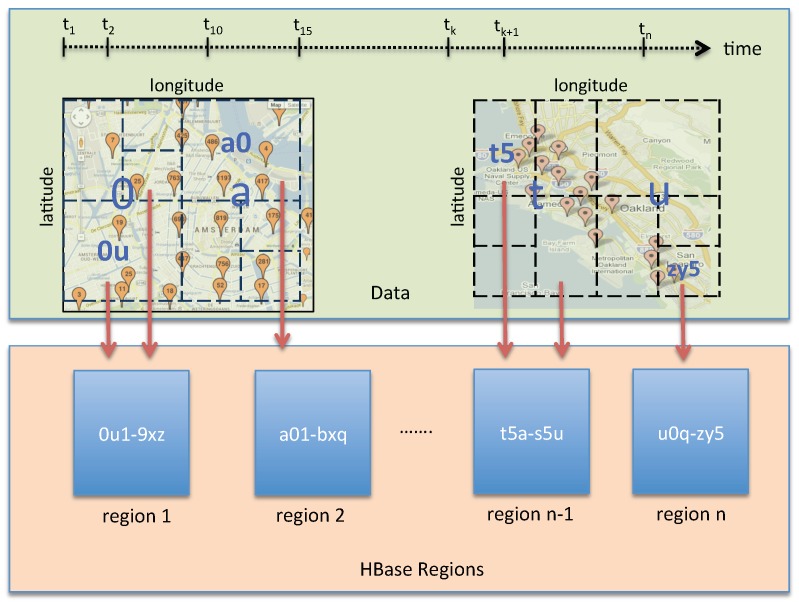
Spatio–temporal data partitioning strategy.

Although HBase already includes partitioners, they do not make use of the spatio–temporal characteristics. In our approach, we partition the tsdb table into a pre-configured number of regions. Each region is assigned with a unique range of geohash prefix. Figure 9 illustrates our spatio–temporal data partitioning strategy. In this figure, region 1 is assigned with a range [0u1–9xz], indicating that all data records that have rowkey prefixes within the range of [0u1–9xz] will be stored in region 1. By applying the spatio–temporal partitioning strategy, we ensure that all sensor data that are near to each other in time and space will be stored in the same partition. As demonstrated later in our experiments in Section 7, with the help of this strategy, the EAGLE engine is able to quickly locate what partitions actually have to be processed for a query. For example, a spatial intersect query only has to check the items of partitions where the partition bounds themselves intersect with the query object. Such a check can decrease the number of data items to process significantly and thus, also reduce the processing time drastically.

## 5. System Implementation

In this section, we will present in details the EAGLE’s implementation based on the architecture presented in Section 3.

### 5.1. Indexing Approach

In order to ensure efficient execution of spatio–temporal queries in EAGLE, we must provide a means to extract and index portions of the sensor data based on spatial, temporal and text values. In this section, we firstly present how to define triple patterns for extracting spatial, temporal and text data. After that, we describe in detail the indexing schemes for each aspect of sensor data.

#### 5.1.1. Defining Triple Patterns for Extracting Spatio–Temporal Data

As mentioned earlier, spatio–temporal and text data included in input RDF sensor data are extracted if their data graph matches the pre-defined triple patterns. In EAGLE, we support several common triple patterns already defined in a set of widely-used ontologies for annotating sensor data, such as GeoSPARQL, OWL-Time, WGS84, SOSA/SSN. For example, we support the GeoSPARQL pattern (?s geo:asWKT ?o) for extracting spatial data. In addition to the commonly used patterns, the ones with user-customized vocabularies are also allowed in our engine. All triple patterns for extracting spatio–temporal data are stored in the data analyzer component. In EAGLE’s implementation, these patterns can be defined by either in the configuration files or via provided procedures. Listing 4 illustrates an example of using configuration file to define triple patterns for extracting spatial data. In this example, the two defined predicates, wgs84:lat/wgs84:long and geo:asWKT, are used to extract the spatial information from input RDF graphs. To reduce the learning efforts, our configuration file syntax fully complies with the Jena assembler description syntax [40].

The process to define triple patterns for extracting temporal data is a bit more complicated. As mentioned in Section 4.3, in addition to the observation value and its timestamp, our OpenTSDB rowkey scheme also stores other attributes such as the full geohash prefix, observed property, sensor URI, etc. Therefore, the triple patterns for extracting this additional information should also be defined. An example of defining triple patterns for extracting temporal data is illustrated in Listing 5.


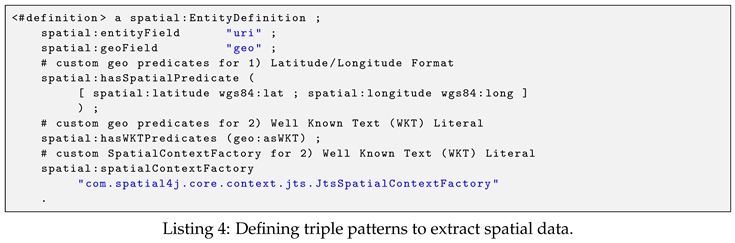



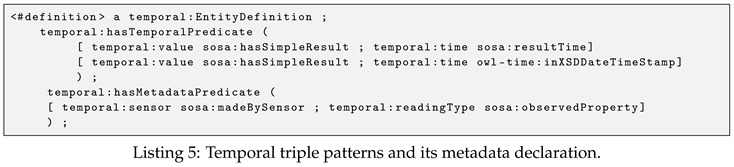


In the above example, triple patterns for extracting temporal value are defined as an instance of the temporal:EntityDefinition. Its property, temporal:hasTemporalPredicate, indicates the RDF predicates used in the matching process of temporal data and timestamp. For example, a pair (temporal:value sosa:hasSimpleResult) denotes that the object of triples that match pattern (?s sosa:hasSimpleResult ?o) will be extracted as a temporal value. Similarly, a pair (temporal:time sosa:resultTime) specifies the predicate sosa:resultTime used for extracting the timestamp. Finally, triple patterns that describe additional information are defined under the temporal:hasMetadataPredicate property, i.e., the sensor URI (extracted by sosa:madeBySensor) and the reading type (extracted by sosa:observedProperty). It is worth mentioning that the current generation of EAGLE supports only the time instant. Time interval support will be added in the next version.

#### 5.1.2. Spatial and Text Index

We store spatial and text data in the ElasticSearch cluster. Therefore, we first need to define the ElasticSearch mappings for storing these data. In ElasticSearch, mapping is the process of defining how a document, and the fields it contains, are stored and indexed.

The ElasticSearch geo mapping for storing spatial objects is shown in Listing 6. In this mapping, the uri field stores the geometry IRI, and the full_geohash field stores the 12-bit geohash string of the sensor location. Similarly, the ElasticSearch mapping for storing text value is shown in Listing 7.

In EAGLE, we support both bulk and near real-time data indexing. Bulk index is used to import data that are stored in files or in the triple store. For this, we provide a procedure build_geo_text_index(). After having the ElasticSearch mappings defined, the build_geo_text_index() is called to construct a spatial index for a given dataset. The pseudo code of this procedure is given in Algorithm 1. In contrast to the bulk index, the near real-time index is used to index the data that are currently streaming to the engine. In this regard, a procedure dynamic_geo_text_index() is introduced to extract spatial and text data from a streaming triple and index them in ElasticSearch. Algorithm 2 describes the pseudo code of the dynamic_geo_text_index() procedure.


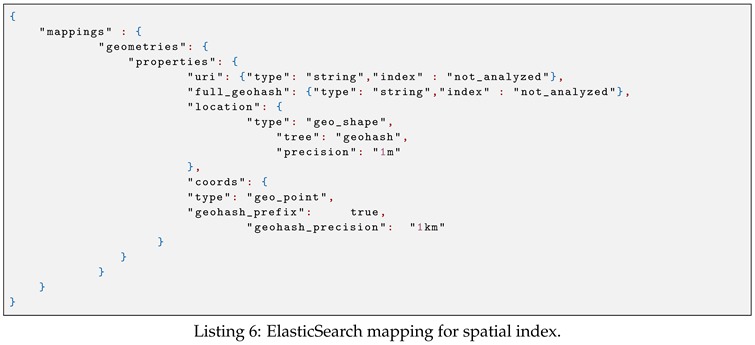



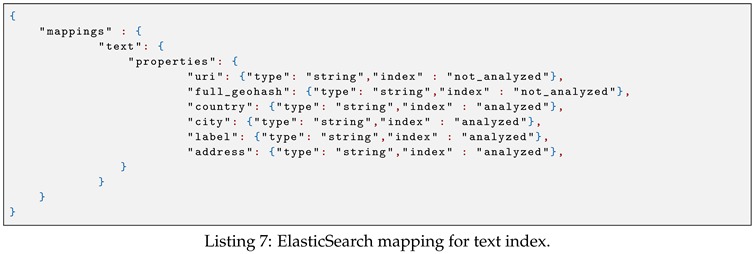


**Algorithm 1:** A procedure that will read a given dataset and index its spatial and text data in ElasticSearch.

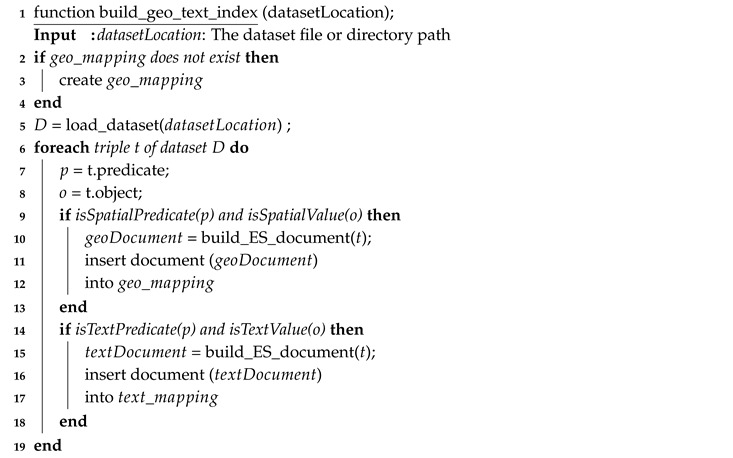



**Algorithm 2:** A procedure that will read a streaming triple and index its spatial and text data in ElasticSearch.

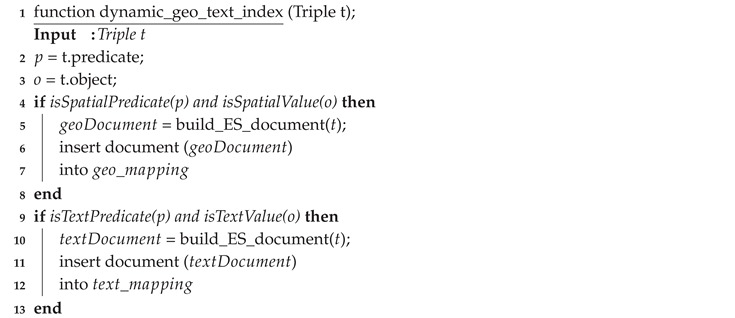



#### 5.1.3. Temporal Index

We provide the procedure, namely build_temporal_index, to construct a temporal index for given sensor observation data. The build_temporal_index procedure is split into three steps, as illustrated in Algorithm 3. The procedure is explained as follows. Firstly, the sensor metadata is loaded into the system memory. This metadata is used later for quickly retrieving information needed for constructing OpenTSDB data row, such as sensor location, observed properties, etc. The loaded metadata can be stored in a key-value data structure such as hashmap, array, etc.

**Figure 10:**
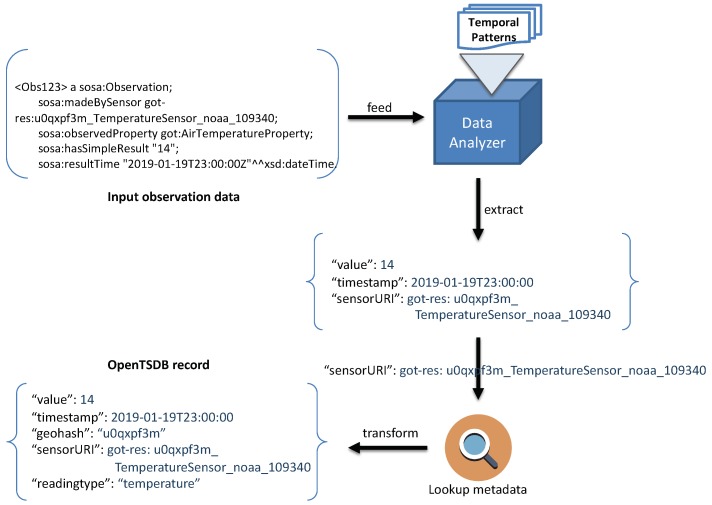
An example of temporal information extraction process.

In the second step, we extract the observation value, its timestamp, and the IRI of source sensor based on the defined triple patterns. After having this information extracted, corresponding observed property and sensor location are then retrieved by querying the loaded metadata in step 1. Thereafter, from the retrieved sensor location, the corresponding geohash prefix is generated via a build_geohash_prefix procedure. The second step is demonstrated in Figure 10.

**Algorithm 3:** A procedure that will read an observation data and index its temporal information in OpenTSDB.

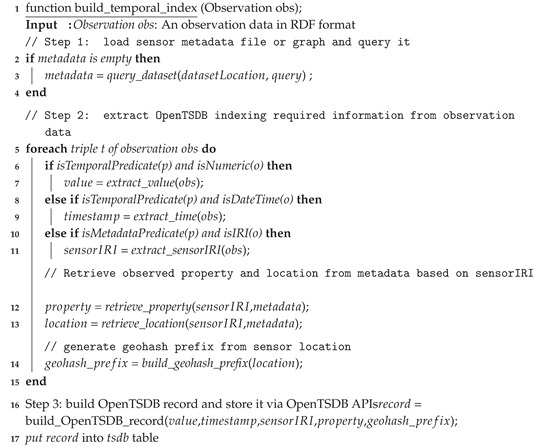



**Figure 11:**
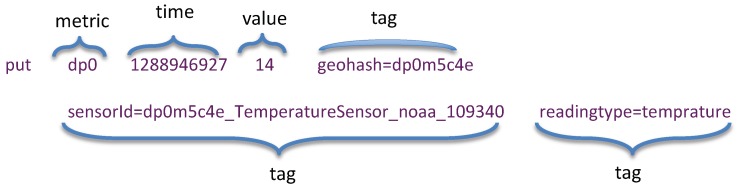
TSDB put example.

The final step is to generate an OpenTSDB data record from the data extracted in the previous steps and store it into OpenTSDB tsdb table. Data are indexed by calling OpenTSDB APIs such as *put* command, REST APIs, etc. Figure 11 illustrates a simple data insert operation in OpenTSDB using *put* command.

### 5.2. Query Delegation Model

Our query execution process of EAGLE is implemented by a query delegation model which breaks the input query into sub-queries that can be delegated to the underlying sub-components such as ElasicSearch, OpenTSBD, and Apache Jena. In this model, a spatio–temporal query can be represented by the SPARQL query graph model (SQGM) [41]. A query translated into SQGM can be interpreted as a planar rooted directed labeled graph with vertices and edges representing operators and data flows, respectively. In SQGM, an operator processes and generates either an RDF graph (a set of RDF triples), a set of variable bindings or a boolean value. Any operator has the properties input and output. The property input specifies the data flow(s) providing the input data for an operator and output specifies the data flow(s) pointing to another operator consuming the output data.

**Figure 12:**
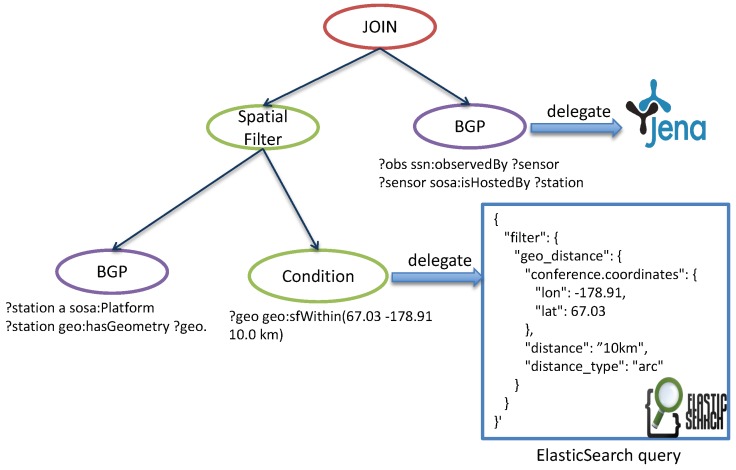
Delegating the evaluation nodes to different backend repositories.

An evaluation process of the graph is implemented by following a post-order traversal, during which the data are passed from the previous node to the next. In this tree, each child node can be executed individually as asynchronous tasks, which can be carried out in different processes on different computers. Therefore, our system delegates some of those evaluation tasks to different distributed backend repositories, which can provide certain function sets, e.g., geospatial functions (by ElasicSearch), temporal analytical functions (by OpenTSDB), BGP matching (by Jena) and achieve the best performance in parallel. Figure 12 shows an example of SQGM tree on which a spatial filter node is rewritten to geospatial query and then delegated to ElasticSearch while the BGP matching query is executed by Jena.

## 6. Query Language Support

In this section, we present our SPARQL query language extensions for querying linked sensor data. We adopt the GeoSPARQL syntax [42] into our proposed extensions for querying topological relations of spatial objects. Furthermore, we also introduce a set of novel temporal analytical property functions for querying temporal data.

### 6.1. Spatial Built-in Condition

Theoretically, a spatial built-in condition is used to express the spatial constraints on spatial variables. As previously mentioned, our proposed SPARQL’s extensions adopt the GeoSPARQL syntax for querying spatial aspect of sensor data. Therefore, in this section, we paraphrase the notion of the spatial built-in conditions that are related to the EAGLE’s implementation. More details of GeoSPARQL built-in condition can be found in [42]. It is important to mention that, within the scope of this paper, we only focus on the qualitative spatial function.

A qualitative spatial function is a Boolean function $f_{s}$, defined as follows:

(1) $$f_{s}:G\times G\to B$$

where *G* is a set of geometries.

In the current version of EAGLE, several topological relations are supported: disjoint, intersect, contains, within. Following the qualitative spatial function definition, we then define a qualitative spatial expression, denoted by “se”:

(2) $$<\mathrm{se}>::=f_{s}\left(g_{1},g_{2}\right)$$

where $g_{1},g_{2}\in G\cup V$. *V* is a set of variables.

A spatial built-in condition is then defined by using the qualitative spatial function, logical connectives ¬,∧,∨:

- If <se> is a qualitative spatial function, then <se> is a spatial built-in condition.
- If $R_{1}$, $R_{2}$ are spatial built-in conditions, then ($\neg R_{1}$), ($R_{1}\vee R_{2}$), and ($R_{1}\wedge R_{2}$) are spatial built-in conditions.

### 6.2. Property Functions

In addition to spatial built-in condition, we also define a set of spatio–temporal and full-text search property functions. By definition, property function is an RDF predicate in SPARQL query that causes triple matching to happen by executing some specific data processing other than usual graph matching. Property functions must have fixed URI for the predicate and can not represent query variables. The subject or object of these functions can be a list.

In our query language support, the property functions are categorized into three types, depending on their function, which are spatial property function, temporal property functions, and full-text search property functions. These three types of property functions are assigned with different URIs (<geo:>, <temporal:>, <text:>). Spatial and temporal property functions are defined as follows.

Drawing upon the theoretical treatments of RDF in [43], we assume the existence of pairwise-disjoint countably infinite sets *I*, *B* and *L* that contain IRIs, blank nodes and literals respectively. *V* is a set of query variables. We denote a set of spatial property functions like $I_{SPro}$. Similarly, let $I_{TPro}$ be a set of temporal property functions. $I_{SPro}$, $I_{TPro}$ and I are also pairwise-disjoint.

A triple that contains a spatial property function is defined in the following form:

(3) $$\left(I\cup B\right)\times I_{SPro}\times \left(I\cup L\cup V\right).$$

**Example** **1.** *Following is the example of spatial property function, namely geo:sfWithin, to find all the ?geo*_1_* objects that are within ?geo*_2_**

$$?{\mathrm{geo}}_{1}\mathrm{geo:sfWithin}?{\mathrm{geo}}_{2}$$

Similarly, the temporal property function is defined as follows:

(4) $$\left(I\cup B\right)\times I_{TPro}\times \left(I\cup L\cup V\right).$$

An example of temporal property function is temporal:avg. For the full-text search property function, we only support one property function, namely text:match. The usages of the property functions will be demonstrated via examples in Section 6.3.

### 6.3. Querying Linked Sensor Data by Examples

This section presents the syntax of our proposed SPARQL’s spatio–temporal extensions through a series of examples involving linked sensor data. The dataset is used throughout the examples is the linked meteorological data described later in Section 7.1.2. The namespaces used in the examples are listed in Appendix A.

**Example** **2.** *(Spatial built-in condition query). Return the IRIs and coordinates of all weather stations that locate in Dublin city. The query is shown in Listing 8.*

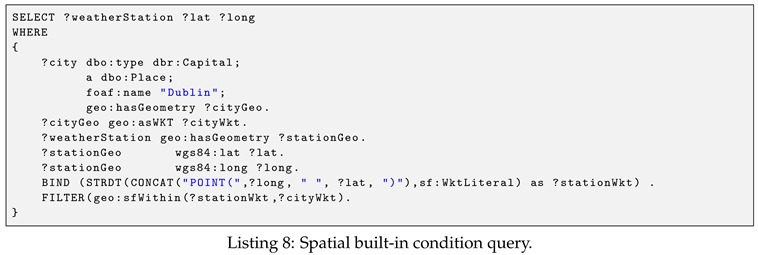

Let us now explain the query syntax by referring to the above example. Recall that our spatial query language adopts GeoSPARQL syntax, hence, all the GeoSPARQL prefixes, as well as its spatial datatypes, remain unchanged. As illustrated in the query, the spatial variables, ?cityWkt and ?stationWkt, can be used in basic graph patterns and refer to spatial literals. Note that, a spatial variable is an object of a spatial predicate in the triple pattern. In this example, the spatial predicate is geo:asWKT defined in [42]. In addition to the basic graph patterns, the spatial variables are also used in the FILTER expression. Similarly to the spatial predicate, the spatial built-in condition in FILTER expression is also assigned with a unique namespace. The current version of EAGLE supports several topological spatial relations such as geo:sfWithin, geo:sfDisjoint, geo:sfIntersects, geo:sfContains.

**Example** **3.** *(Spatial property function query). Given the latitude and longitude position, it retrieves the number of nearest weather stations that are located within 20 miles. The query is shown in Listing 9.*

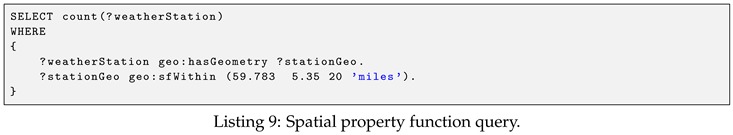

The above query demonstrates the usage of geo:sfWithin property function. When this property function is called, a dedicated piece of code will be executed to find all the geometries locate within an area. The area is specified by these arguments (59.783 5.35 20 ‘miles’). For each spatial object that satisfies the spatial condition, its IRI is bound to the ?stationGeo variable that occurs in the triple representing the property function call. In addition to the default GeoSPARQL syntax of this function, we additionally extend its usage as follows:

$$\begin{array}{c} \mathrm{GeoSPARQL}\;\mathrm{syntax}:<{\mathrm{feature}}_{1}>\mathrm{geo:sfWithin}<{\mathrm{feature}}_{2}>\\ \mathrm{Our}\;\mathrm{extension}:<{\mathrm{feature}}_{1}>\mathrm{geo:sfWithin}\;(<\mathrm{lat}><\mathrm{lon}><\mathrm{radius}>[<\mathrm{units}>[<\mathrm{limit}>]]). \end{array}$$

Table 2 describes the list of spatial property functions that are currently supported in EAGLE. These functions allow the user to specify the query bounding box area by either using the <geo> parameter or using the concrete coordinates via <lat>, <lon>, <latMin>, etc. The <geo> parameter can be a spatial variable or a spatial RDF literal. Similarly, the <units> can be a unit URI or a string value. The supported distance units are presented in Table 3. Finally, the <limit> parameter is to limit the number of results returned by the function.

**Example** **4.** *(Temporal property function query). Return the list of air temperature observation values that are generated by the station <got-res:WeatherStation/gu9gdbbysm_ish_1001099999> from 10th to 15th March 2018. The query is shown in Listing 10.*

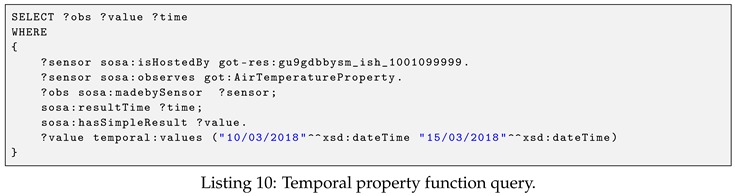

The above query demonstrates the example usage of one of our temporal property functions, called temporal:values. In this query, the property function temporal:values is called to retrieve all the temperature observation values that are generated within a specific time interval. Recall that the prefix <temporal:> is used to represent the temporal property function. Table 4 lists all the supported temporal property functions and their syntax. The usages of these functions will be demonstrated in the following examples.

**Example** **5.** *(Analytical spatio–temporal query). Detection of all wind-speed observation in an area within 40 miles from the center of Ohio City during the time from 10 January to 10 February 2017. The Ohio City center coordinate is (40.417287-82.907123). The query is shown in Listing 11.*

The query above demonstrates the mix of spatial and temporal property functions. The query uses the spatial function, namely geo:sfWithin, to filter all weather stations that locate in the area (40.417287-82.907123 40 ‘miles’). Additionally, it also retrieves the list of wind speed observation values generated by these station with the time constraint.

**Example** **6.** *(Analytical spatio–temporal query). Calculate the daily average windspeed at all weather stations that locate within 20 miles from London city center during the time from 10 to 15 March 2018. The query is shown in Listing 12.*

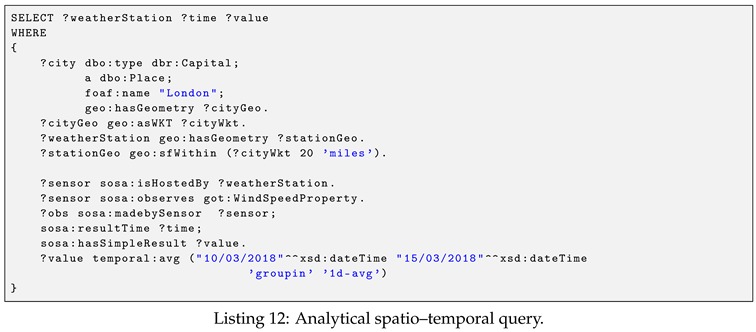

The query demonstrates a complex analytical spatio–temporal query. In this query, we first retrieve the London geometry data by querying the DBPedia dataset. After that, we use the spatial function, namely geo:sfWithin, to query all the stations that locate within 20 miles from London. In the temporal property function used in this query, we demonstrate the usage of the downsampler feature indicated by groupin keyword, and the downsampling aggregation function. Given a brief description, the downsampler feature is our additional temporal query feature which aims to simplify the data aggregation process and to reduce the resolution of data. The data aggregation and data resolution are specified by the downsampling aggregation function, which is formed by <time interval>_<aggregation function>. The <time interval> is specified in the format <size><units> such as 1 h or 30 m. The aggregation function is taken from the list (sum, average, count, min, max). For example, as illustrated in the query, the downsampling aggregation function is 1 d-avg.


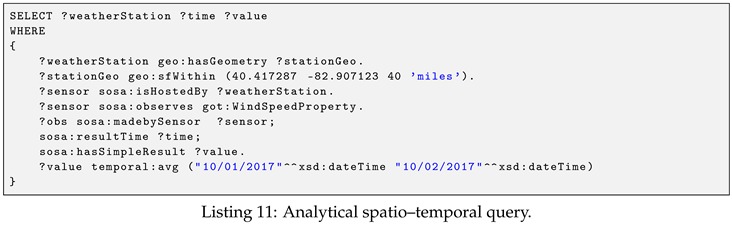


Example usage of downsampler can be described as follows. Let us say that a wind-speed sensor is feeding observation data every second. If a user queries for data over an hour-long time span, she would receive 3600 observation data points, something that could be graphed fairly easily in the result table. However, let us consider the case that the user asks for a full week of data. For that, she will receive 604,800 records, thus, leading to a very big result table. Using a downsampler, multiple data points within a time range for a single time series are aggregated together with an aggregation function into a single value at an aligned timestamp. This way, the number of return values can be reduced significantly.

**Example** **7.** *(Analytical spatio–temporal query). Retrieve the weekly average temperature of area B which has geohash “u0q” in March 2018. This query illustrates the usage of two optional arguments in the temporal property functions, namely geohash and observableProperty. The query is shown in Listing 13.*

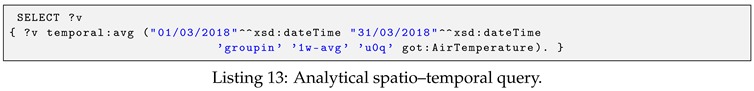

**Example** **8.** *(Full-text search query). Retrieve the total number of observation for each observed property of places that match a given keyword ‘Cali’. The query is shown in Listing 14.*

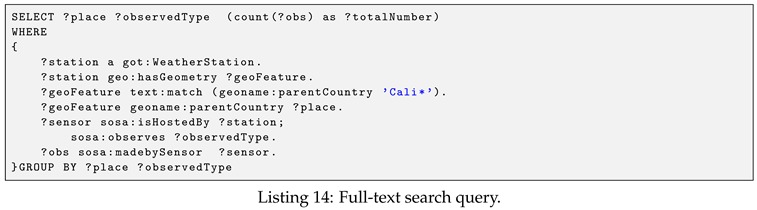

The above query demonstrates the usage of full-text search feature via the text:match property function. The text:match syntax is described as follows:

$$\begin{array}{l} <\mathrm{subject}>\mathrm{text}:\mathrm{match}\;(<\mathrm{property}>‘\mathrm{query}\;\mathrm{string}’<\mathrm{limit}>) \end{array}$$

In the text:match function syntax, the <subject> implies the subject of the indexed RDF triple. It can be a variable or an IRI. The <property> is an IRI, of which the literal is indexed, e.g., rdfs:label and geoname:parentCountry. The ‘query string’ is the query string fragment following the Lucence syntax (https://lucene.apache.org/core/2_9_4/queryparsersyntax.html). For example, the parameter ‘Cali*’ is to select all the literals that match prefix “Cali”. The optional limit limits the number of literals returned. Note that, it is different than the number of total results the query will return. When a limit is specified in the SPARQL query, it does not affect the full-text search, rather, it only restricts the size of the result set.

## 7. Experimental Evaluation

In this section, we present a rigorous quantitative experimental evaluation of our EAGLE implementation. We divide the presentation of our evaluation into different sections. Section 7.1 describes the experimental setup which includes the platform and software used, datasets, and queries descriptions. Section 7.2 presents the experimental results. In this section, we compare the data loading throughput and query performance of EAGLE against Virtuoso, Apache Jena and GraphDB. We also discuss the performance differences in EAGLE when applying our data partitioning strategy, described in Section 4.3. Finally, we evaluate EAGLE’s performance on a Google Cloud environment to demonstrate its elasticity and scalability as regards data loading and query performance. The strengths and weaknesses of the EAGLE engine are discussed in Section 7.3.

### 7.1. Experimental Settings

#### 7.1.1. Platform and Software

To demonstrate EAGLE’s performance and scalability, we evaluate it on a physical setup and a cloud setup. It is worth mentioning that our physical setup is dedicated to a live deployment of our GraphOfThings application at http://graphofthings.org which has been ingesting and serving data from more than 400,000 sensor data sources since June 2014. We compare EAGLE’s performance against Apache Jena v3.12, Virtuoso v7 and GraphDB v8.9 (former OWLIM store [27]). Among them, Jena represents the state-of-the-art in terms of a native RDF store, Virtuoso is a widely used RDF store backed by RDBMS, and GraphDB is a clustered RDF store that has recently supported spatial querying.

We deployed Apache Jena and Virtuoso v7 on a single machine with the same configuration as in our physical setup below. For EAGLE, we installed ElasticSearch v7 and OpenTSDB v2.3 for both the physical and cloud setups. Similarly, we also installed the GraphDB v8.9 on all setups.

Physical setup: we deployed a physical cluster that consists of four servers running on the shared network backbone with 10 Gbps bandwidth. Each server has the following configuration: 2x E5-2609 V2 Intel Quad-Core Xeon 2.5GHz 10MB Cache, Hard Drive 3x 2TB Enterprise Class SAS2 6Gb/s 7200RPM - 3.5” on RAID 0, Memory 32GB 1600MHz DDR3 ECC Reg w/Parity DIMM Dual Rank. One server is dedicated as a front-end server and to coordinating the cluster, and the other three servers are used to store data and run as processing slaves.

Cloud setup: the cloud setup was used to evaluate the elasticity and scalability of the EAGLE engine. We deployed a virtual cluster on Google Cloud. The configuration of the Google Cloud instances we use for all experiments is the “n1-standard-2” instance, i.e., 7.5 GB RAM, one virtual core with two Cloud Compute Units, 100 GB instance storage, and an Intel Ivy Bridge platform. In this evaluation, we focused more on showing how the system performance scales when increasing the number of processing nodes, rather than serving as a comparison of its performance with the physical cluster.

#### 7.1.2. Datasets

Our experimental evaluations are conducted over the linked meteorological dataset which is described in [18,44]. The dataset consists of more than 26,000 meteorological stations allocated around the world and covers various aspects of data distribution. The window of archived data is spread over 10 years, from 2008 to 2018. It has more than 3.7 billion sensor observation records which are represented in the SSN/SOSA observation triple layout (seven triples/records). Hence, the data contains approximately 26 billion triples if it is stored in a native RDF store.

Additionally, in order to give a more practical overview of the engine, we evaluated it on even more realistic datasets, especially ones consisting of both spatial and text data. To meet such requirements, we select several datasets from GoT data sources [18]. In particular, to evaluate the spatial data loading throughput, in addition to the sensor station location, we also import the transportation dataset which contains 360 million spatial records. These records were collected from 317,000 flights and 20,000 ships during the time 2015–2016. Similarly, for the text data loading evaluation, we import a Twitter dataset that consists of five million tweets. The detailed statistics of all the datasets used for our evaluations are listed in Table 5.

#### 7.1.3. Queries

We have selected a set of 11 queries that were performed over our evaluation datasets. In general, our queries aim to check the engine processing capability with respect to their provided features for querying linked sensor data. Because the standard SPARQL 1.1 language does not support spatio–temporal queries nor full-text search queries, some RDF stores have to extend the SPARQL language with their own specific syntax. Therefore, some of these queries need to be rewritten so they can be compatible with the engine under test.

We summarize some highlighted features of the queries as follows: (i) if the query has an input parameter; (ii) if it requires geospatial search; (iii) if it uses a temporal filter; (iv) if it uses full-text search on string literals; (v) if it has a group-by feature; (vi) if the results need to be ordered via an order-by operator; (vii) if the results are using the limit operator; (viii) the number of variables in the query; and (ix) the number of triple patterns in the query. The group-by, order-by, and limit operators impact on the effectiveness of the query optimization techniques used by the engine (e.g., parallel unions, ordering or grouping using indexes, etc.), and the number of variables and triple patterns give a measure of query complexity. This summary of highlighted features and their SPARQL representations are described in Appendix A and Appendix B, respectively.

### 7.2. Experimental Results

#### 7.2.1. Data Loading Performance

We evaluated EAGLE’s performance with respect to data loading throughput on our physical setup and compared it to the state-of-the-art systems. Benchmark data were stored in files and imported via bulk loading. Unlike the general performance comparisons that only focus on triple data loading performance, we measure separately the loading performance of spatial, text and temporal data. The loading speed was calculated via the number of objects that can be indexed per second, instead of the number of triples. This evaluation helped us to have a better understanding of the indexing behavior of the test engines for specific types of data such as geospatial and text.

##### Spatial Data Loading Performance With Respect to Dataset Size

**Figure 13:**
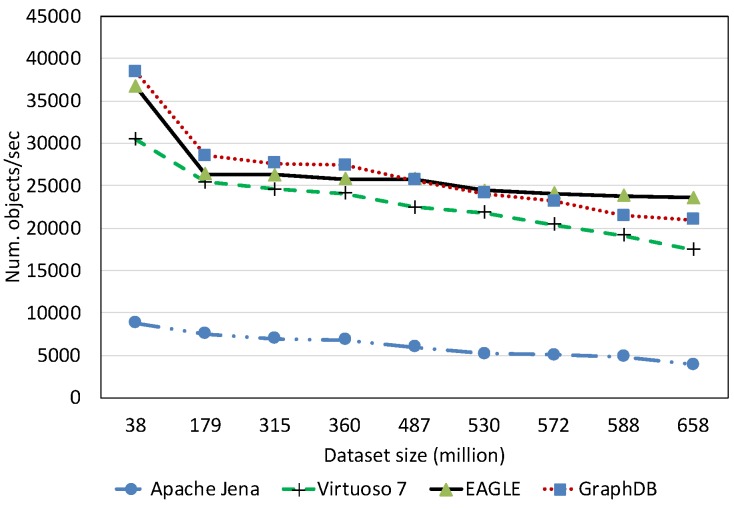
Average spatial data loading throughput.

**Figure 14:**
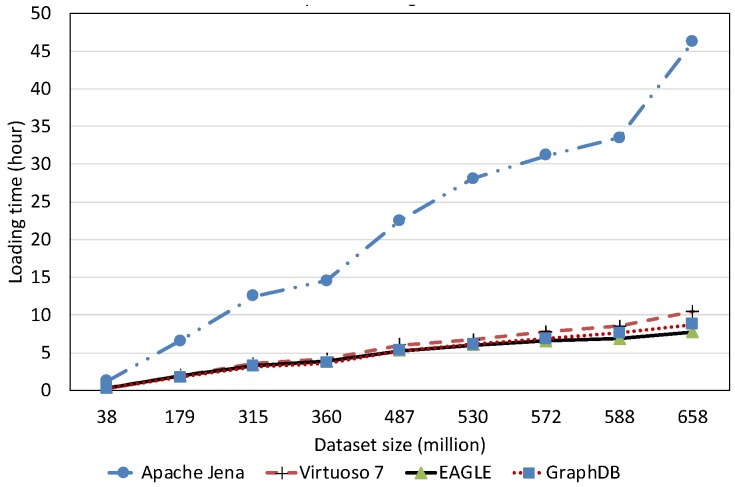
Spatial data loading time.

Figure 13 depicts the average spatial data loading speed of the four evaluated RDF systems, with respect to various dataset sizes. The data loading time is shown in Figure 14. Overall, the results reveal that the increase in the data size can significantly affect the loading performance of all systems. Among them, Apache Jena has the worst performance. The average data loading speed is below 10,000 obj/s for all dataset sizes, slower than the other systems. Moreover, it takes almost two days (46.23 h) for loading 658 million spatial data objects. The data loading performance of EAGLE and GraphDB are very close, followed by Virtuoso. For example, EAGLE loads 658 million spatial objects in 7.74 h. Its average throughput is 23,620 obj/s. In the meantime, GraphDB is one hour behind, resulting in 8.72 h and the average speed is 20,960 obj/s. Virtuoso achieves a speed of 17,500 obj/s. The slower insert speed of Virtuoso and Jena can be explained by the limit of single data loading processes in these systems, which are deployed on a single machine. This sharply contrasts with the parallel data loading processes supported by the distributed back-end DBMS in EAGLE (ElasticSearch and OpenTSDB) and GraphDB.

We also learned that in the beginning, EAGLE performs slightly behind GraphDB in the case of loading a small dataset (<350 million). We hypothesize that this is due to several reasons such as load imbalance, increased I/O traffic and platform overheads in EAGLE. However, for loading larger datasets, this comparison result is reversed and the spatial data loading performance of GraphDB is slower than ours. This highlights the capabilities of our system for dealing with the “big data” nature of sensor data.

##### Text Data Loading Performance With Respect to Dataset Size

**Figure 15:**
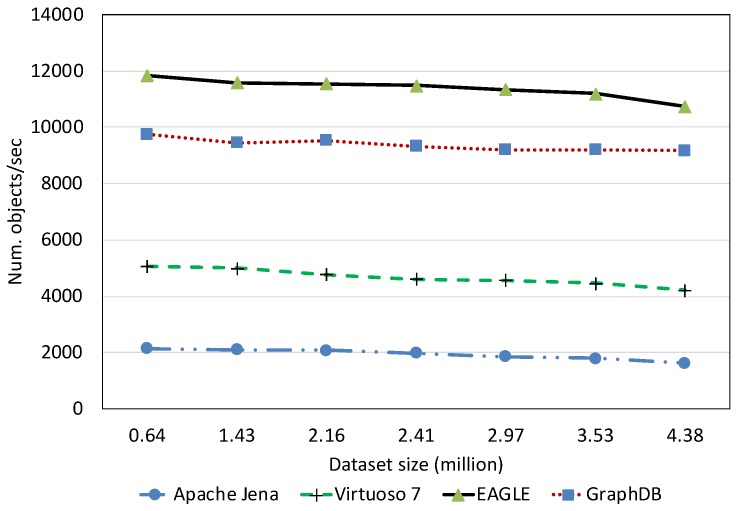
Average full-text indexing throughput.

**Figure 16:**
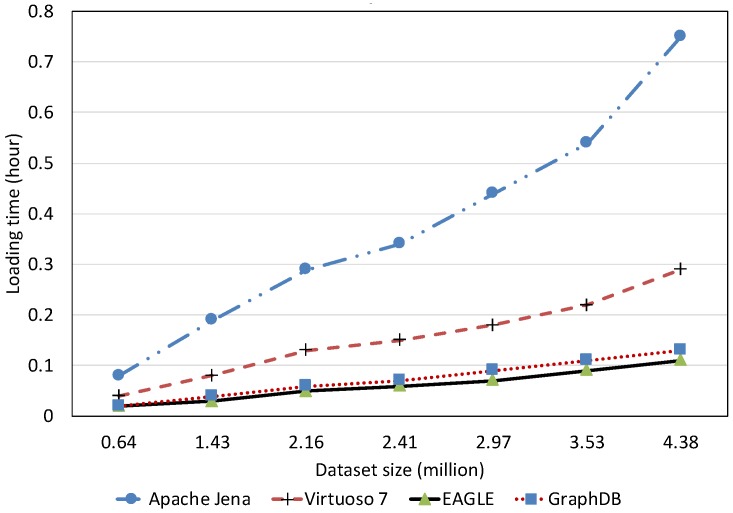
Text data loading time.

To evaluate the text data loading performance, we load the Twitter dataset that consists of five million tweets in the RDF format. The loading speed and loading time are reported in Figure 15 and Figure 16, respectively. According to the results, EAGLE outperforms the other systems. We can see in Figure 15 that its loading speed is just lightly affected by the data size increase. The highest speed EAGLE can reach is 11,800 obj/s for loading 0.64 million tweets. We attribute this to the outstanding performance of EAGLE’s databases, namely ElasticSearch, which is originally a document-oriented database. In the case of loading the same data size, GraphDB is slower than EAGLE. Its average speed is 9700 obj/s. Virtuoso and Jena follow at two and five times slower than EAGLE, respectively.

In comparison with the spatial data loading performance, the text data loading speed of EAGLE is much slower. This is reasonable because in order to index the text data, the system needs to analyze the text and break it into a set of sub-strings. Consequently, this requires more computation and resource consumption, and hence, increases the overall loading time.

##### Temporal Data Loading Performance With Respect to Dataset Size

We evaluated the temporal data loading performance by importing our 10 years of historical linked meteorological data. In this evaluation, we also measured the performance of EAGLE when disabling the spatio–temporal partitioning feature, denoted by EAGLE-NP. Instead of loading the entire temporal dataset, we terminated the loading process at 7.78 billion triples due to the long data loading time, and some of the evaluated systems stop responding.

**Figure 17:**
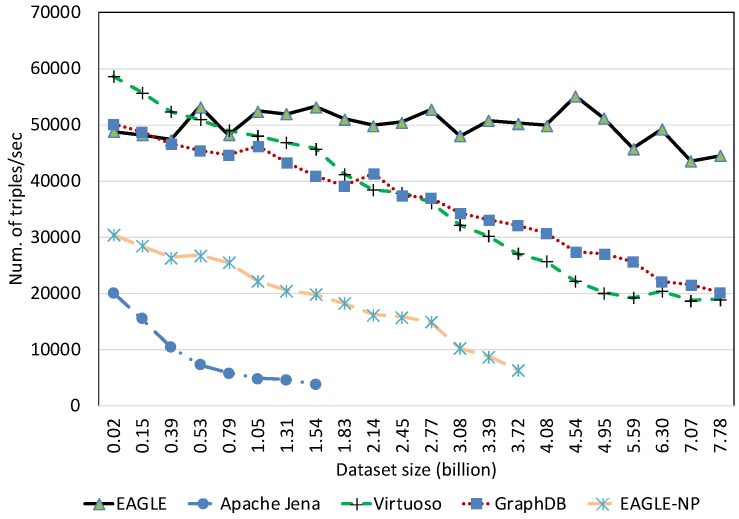
Average temporal indexing throughput.

**Figure 18:**
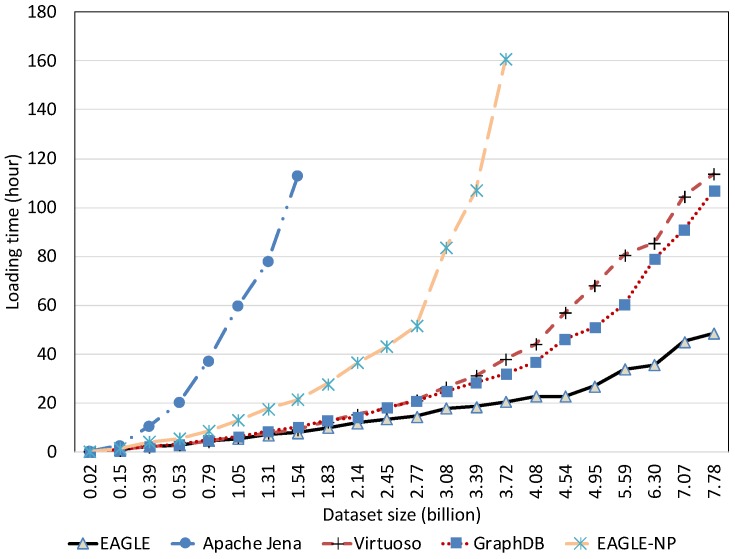
Temporal data loading time.

The results in Figure 17 and Figure 18 draw our attention to the performance of all systems when loading the small dataset. Regardless of the poor performance of Apache Jena, it is apparent that Virtuoso had better loading performance than EAGLE and GraphDB in the case of loaded data sizes under 100 million data points. A possible explanation for this phenomena is the communication latency of the distributed components in GraphDB and EAGLE. More precisely, in these distributed systems, the required time for loading data, plus the time for coordinating the cluster and the network latency are more than the data loading time in Virtuoso. Nevertheless, the difference is acceptable and we believe EAGLE is still applicable for interactive applications that only import a limited amount of data.

Another interesting finding is that our system performs differently if the spatio–temporal partitioning strategy is disabled. In this case, the highest insert speed that EAGLE-NP can achieve is 30k obj/s. However, this speed drops dramatically with the growth of the imported data. Moreover, we also observe that EAGLE-NP stops responding when the data size reaches 3.72 billion records, as depicted in Figure 17 and Figure 18. Looking at the system log files, we attribute this to a bottleneck in performance that happens with the OpenTSDB tsdb table. As previously explained in Section 4.3, if the spatio–temporal data partitioning is disabled, the tsdb table is not pre-split, thus, there is only one region of this table that is initialized. In this case, data are only inserted into this region. As a result, when the I/O disk writing speed cannot adapt to a large amount of fed data, the bottleneck phenomena happens.

The efficiency of EAGLE is demonstrated when applying our proposed spatio–temporal data partitioning strategy. It is even more explicit in the case of loading a large dataset. As evidenced in Figure 17, unlike the others, the average insert speed of EAGLE almost remains horizontal when the number of data instances increases. In particular, the highest speed that EAGLE can reach is 55,000 obj/s, and there is no significant difference when the number of data points rises from 0.02 to 7.78 billion. Moreover, for loading 7.78 billion temporal triples, EAGLE took only 48.51 h. However, in the same case, GraphDB and Virtuoso need 106.97 h and 113.71 h, respectively. The better rank of EAGLE is attributed to our data partitioning strategy in which we pre-split the tsdb table into multiple data regions in advance of the data loading operation. Because each region is assigned with a range of geohash prefixes, data that has different geohash prefixes managed by different regional servers can be inserted in parallel, resulting in a significant increase in terms of data loading performance. However, when the amount of data stored in a pre-split region reaches the given threshold capacity, the region will be re-split automatically. Together with the splitting process, all related data has to be transferred and distributed again. This step will cause additional cost and will affect the system performance. This explains the slight fluctuation of our system insert speed in Figure 17 during the data loading process.

#### 7.2.2. Query Performance with Respect to Dataset Size

This experiment is designed to demonstrate the query performance of all evaluated systems with respect to different data aspects and dataset size. In this experiment, for each query, we measure the average query execution time by varying the dataset imported. In order to give a more detailed view on query performance, based on the query complexity, we group the test queries into several main categories: spatial query, temporal query, full-text search query, non-spatio–temporal query, and mixed query. These query categories are described in Table 6.

To conduct a precise performance comparison, we load different datasets that correspond to the query categories. For example, our spatial datasets are used to evaluate the spatial query performance while the sensor observation dataset is for queries that require a temporal filter. For the non-spatio–temporal queries, we use the static dataset that describes the sensor metadata. It is important to mention that our data partitioning approach is only applied for temporal data stored in OpenTSDB and does not explicitly affect the spatial and full-text search query performance in ElasticSearch. Therefore, in the experiments for spatial and full-text search query performance, the performances of EAGLE and EAGLE-NP are not differentiated.

##### Non-Spatio–Temporal Query Performance

**Figure 19:**
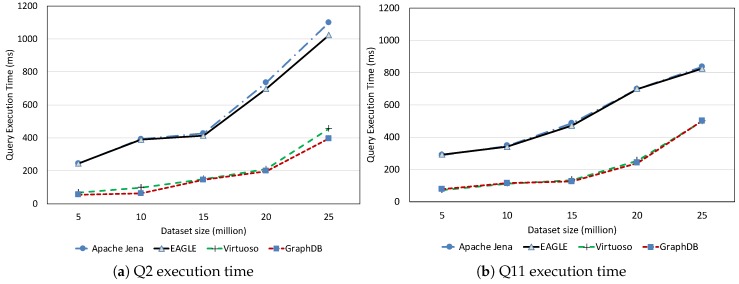
Non spatio–temporal query execution time with respect to dataset size.

We first evaluated the performance of non-spatio–temporal queries, which were Q2 and Q11. These were the standard SPARQL queries which only query on the semantic aspect of sensor data and have neither spatio–temporal computation nor full-text search. The average query execution times are plotted in Figure 19. The results demonstrate the close performance of EAGLE and Apache Jena in regard to non-spatio–temporal queries. This is explained by the use of similar processing engines. In fact, the SPARQL query processing components in EAGLE are extended from Apache Jena ARQ with some modifications. Meantime, Virtuoso and GraphDB prove their reputations in SPARQL query performance by being faster then EAGLE. However, the difference is still acceptable, in the order of ms.

##### Spatial Query Performance

**Figure 20:**
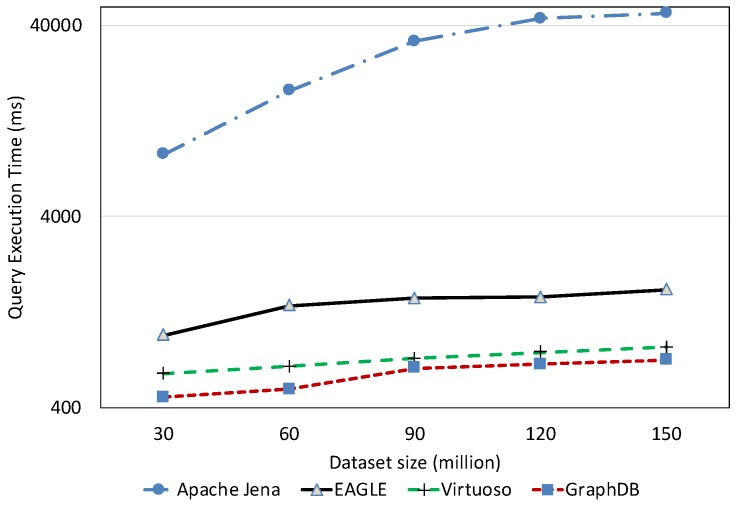
Q1 execution time with respect to spatial dataset size (in logscale).

Figure 20 depicts the query execution time of the spatial query, which is represented by Q1, with respect to varying spatial dataset size. According to the evaluation result, we find that Apache Jena performs poorly. Its spatial query performance linearly increases with increments of the loaded data. In contrast, Virtuoso, GraphDB, and EAGLE perform closely and are weakly influenced by the data size. GraphDB is recognised as having good performance, followed by Virtuoso. Compared to these systems, EAGLE is slightly slower, only in the order of ms. A possible reason could be the overhead of the join operation between the BGP matching and the spatial filter results. Note that, parallel join operations are not yet supported in EAGLE and have to be performed locally in a single thread.

##### Full-Text Search Query Performance

**Figure 21:**
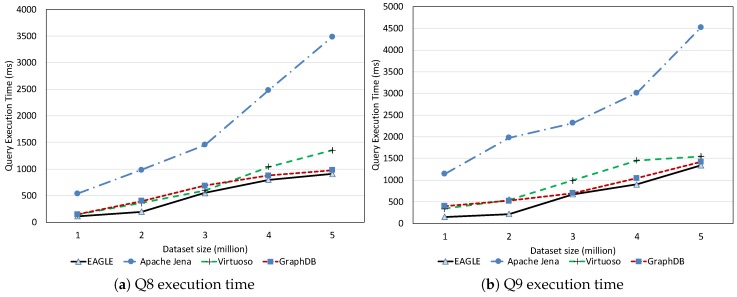
Text-search query execution times with respect to dataset size.

In the following, we discuss the performance of the full-text search queries (Q8, Q9) for the test systems. The evaluation results are reported in Figure 21. Despite the impressive query execution time of GraphDB and Virtuoso, which are generally less than 500 ms for both Q8 and Q9, EAGLE is still slightly faster. This is again thanks to the outstanding performance of ElasticSearch on full-text search queries. Note that, although Apache Jena, GraphDB and ElasticSearch support full-text search through the use of Lucene, ElasticSearch is notable for having a better optimization.

##### Temporal Query Performance

**Figure 22:**
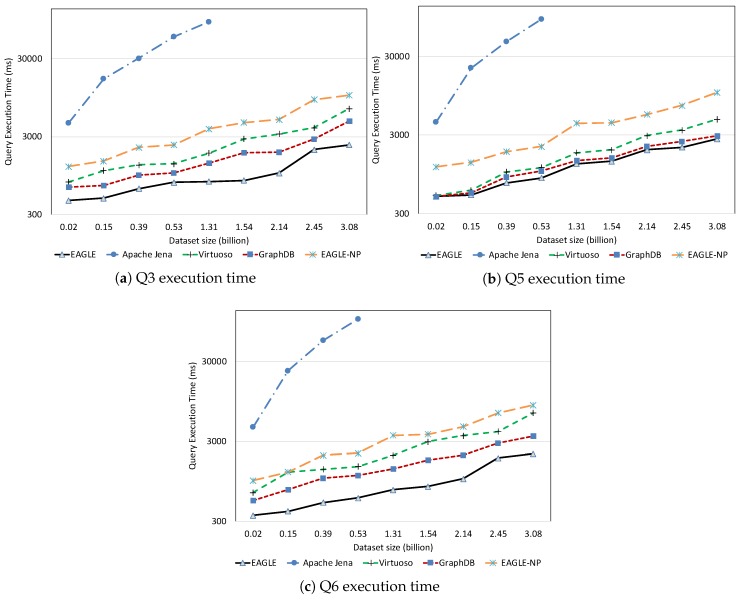
Temporal queries execution time with respect to dataset size (in logscale).

The temporal query performance is evaluated over our historical meteorological observation data. Figure 22 presents the query execution time of all systems with respect to observation dataset size. It is apparent that query performance is affected by an increase in the amount of data. For Apache Jena, along with a linear increase in the query execution time, we also notice that it is only able to run up to a certain amount of data. As we can see in the figure, it could ideally execute queries with datasets under 0.5 billion data points. However, when executing these queries on a dataset which is over 1.31 billion data points, Apache Jena stops responding. The performances of EAGLE and EAGLE-NP are significantly different. For example, when executing over a dataset of 0.53 billion data observations, if the spatio–temporal data partitioning strategy is not applied, the average execution time of Q6 in EAGLE-NP is 2147 (ms). Meanwhile, if the data partitioning strategy is enabled, EAGLE takes only 589 (ms) to execute the same query, resulting in it running four times faster. Furthermore, we also see a better performance of the EAGLE system in comparison with Virtuoso and GraphDB. The explanations for this performance could be: (1) the effectiveness of our data partitioning strategy so that the engine can quickly locate the required data partition and then organize the scans for a large number of data rows; (2) the power of OpenTSDB query functions that we rely on, especially for data aggregation.

##### Mixed Query Performance

**Figure 23:**
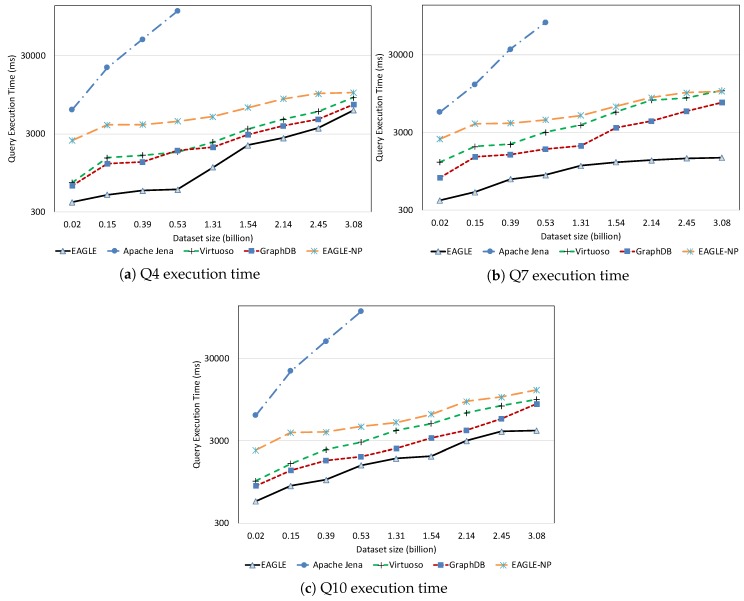
Mixed queries execution time with respect to dataset size (in logscale).

Another aspect to be considered is the performance of the analytics-based queries that require the mixing of spatial, temporal computations or full-text search (Q4, Q7, Q10). For these queries, we increase the query timeout to 120 (s) due to their high complexities. The evaluation results are shown in Figure 23. Apache Jena undergoes time outs for all queries when the loaded data size is over 0.5 billion. Another fact that can be clearly observed is that EAGLE is orders of magnitude faster than the others. This is demonstrated by the case of Q7. Note that, this query implies a heavy computation on both spatial and temporal data. Additionally, it also requires that thhe results have to be ordered by time. As shown in Figure 23b, for executing a query over the dataset with 3.08 billion records, EAGLE performs Q7 much better (1420 ms), follows by GraphDB (7289 ms) and Virtuoso (10,455 ms). There can be several reasons for our impressive performance: (1) The effectiveness of the OpenTSDB time-series data structure such that data are already sorted by time during the loading process. Consequently, in the EAGLE system, for the query that has an order-by operator on date-time, the ordering operation cost, in this case, is eliminated. (2) The second reason again sheds light on the success of our data partitioning strategy and our row-key design so that the time cost for locating the required data partition and the data scan operation is significantly minimized.

#### 7.2.3. Query Performance with Respect to Number of Clients

This experiment is designed to test the concurrent processing capability of EAGLE in a scenario where the system has to deal with a high volume of queries which are sent from multiple users. Rather than serving as a comparison with other stores, this experiment only focuses on analyzing EAGLE’s query processing behavior when receiving concurrent queries from multiple clients. This experiment is performed as follows. In the first step, a dedicated script is built to randomly select and send queries to the system. The query parameters are also randomly generated. In the second step, we perform measurement runs with 10, 100, 250, 500 and 1000 clients concurrently. Finally, for each query, the query execution times are summarized to compute the average value.

**Figure 24:**
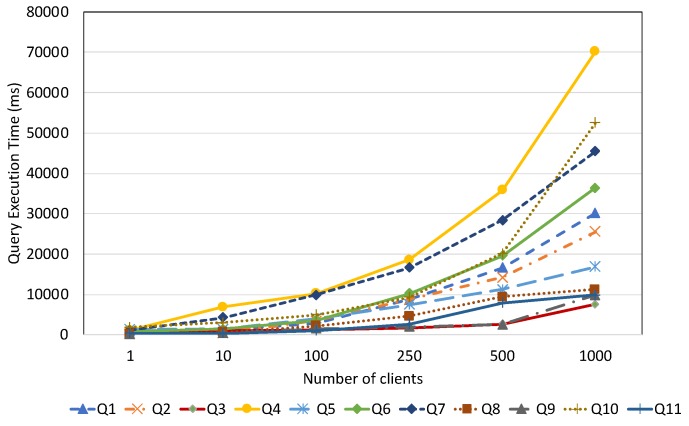
Average query execution time with respect to number of clients.

Figure 24 reports the evaluation results. In general, the execution time for all queries linearly rises when more clients are added. It can be clearly observed that, when the number of clients increases from 250 to 1000, the query execution time increases dramatically. Firstly, this is due to the growing workload applied to the system. Secondly, by deeply analyzing the query cost breakdown, another possible reason is the inefficiency of our query plan cache mechanism. According to our observation, the query cache only works for the non-spatio–temporal queries. However, for the duplicated queries that share the same spatial, temporal and full-text filters, instead of reusing the cached query plan, the query optimizer has to re-generate a new query execution plan. For example, if there are 100 query instances of Q4 that have been sent from 100 clients, the query optimizer has to re-generate the query execution plan for Q4 for 100 times. Obviously, this leads to a dramatic increase for the total query execution time of Q4. As previously mentioned, the EAGLE’s query processing engine has been implemented by extending the widely-known query engine, Jena ARQ, thus, its query cache is identical to the one in Jena ARQ. Unfortunately, the original one was only developed for standard SPARQL queries and does not work for the spatio–temporal queries. Moreover, we also learned that Jena ARQ’s query cache does not work correctly with queries that share similar query patterns but different literals. This is also the case for our tested query patterns, in which the literals are randomly generated. We address this issue by proposing a novel learning approach for spatio–temporal query planning, that is described in [33].

#### 7.2.4. System Scalability

In this experiment, we measure how EAGLE’s performance scales when adding more nodes to the cluster. We vary the number of nodes in the Google Cloud cluster with 2, 4, 8, 12 nodes, respectively.

**Figure 25:**
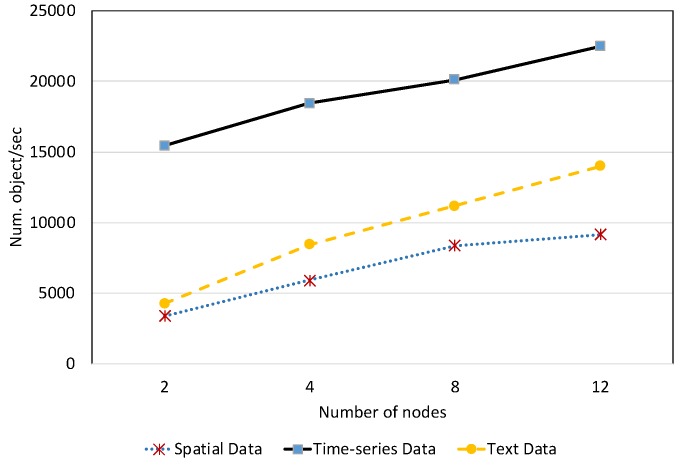
Average index throughput by varying number of cluster nodes.

Figure 25 presents the average loading throughput of spatial, temporal and text data when increasing the number of nodes. The results reveal that the index performance linearly increases with the size of the cluster. This is because scaling out of the cluster causes the working data that needs to be indexed on each machine to be small enough to fit into main memory, which dramatically reduces the required disk I/O operations.

**Figure 26:**
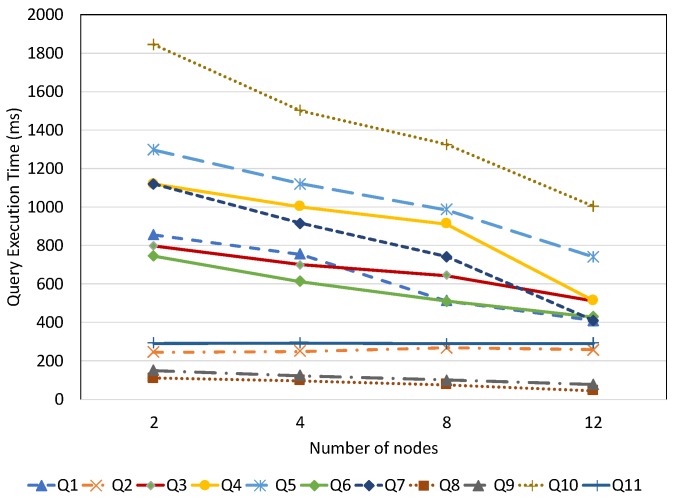
Average query execution time by varying number of cluster nodes.

In the following, we look at the query performance evaluation results shown in Figure 26. According to the results, the query execution times of Q2 and Q11 remain steady and are not affected by the cluster size. This is due to the fact that these queries are non-spatio–temporal queries and only query on the static dataset. Recall that, we store the static dataset on centralized storage (Apache Jena TDB), which is not scalable and is hosted on a single machine. Queries on static data are only executed on this machine. Therefore, it is understandable that, for the non-spatio–temporal queries being executed over the same dataset, scaling out of the cluster has no effect on their performance.

Unlike Q2 and Q11, the performance of other queries scales perfectly with the cluster size. The results indicate that EAGLE has a considerable decrease in query execution time for mixed queries (Q4, Q7, Q10). Meanwhile, other queries have a slightly decreased query execution time. A representative example of mixed queries to demonstrate the scalability of EAGLE is Q4. This query required a heavy spatio–temporal computation on a large number of historical observation data items for a given year. However, along with the scaling out of the cluster, the amount of data processing for this query on each node was also reduced significantly. This explains the rapid drop in the query execution time from 1120 (ms) to 514 (ms) for Q4 when the cluster size scales out from two to 12 nodes, respectively.

### 7.3. Discussion

We have presented an extensive quantitative evaluation of EAGLE’s implementation and conducted a comparison with a top-performing RDF store running on a single node as well as a clustered RDF store. To conduct a precise performance comparison, we measure separately the loading performance of spatial, text and temporal data. The experimental results show that EAGLE performs better than other tested systems in terms of spatio–temporal and text data loading performance. For query performance, we have learned that EAGLE is highly efficient for queries that require heavy spatio–temporal computations on a large amount of historical data. However, it is slightly behind Virtuoso and GraphDB for non-spatio–temporal queries. This is understandable, as improving query performance on semantic data is not our main target.

Another fact that should be highlighted is the effectiveness of our spatio–temporal partitioning strategy and OpenTSDB row-key scheme. This is evidenced by the evaluation results so that EAGLE has outstanding performance when applying the partitioning strategy. In the case where no partitioning strategy is used, it performs poorly and stops responding at a certain dataset size.

For the scalability test, EAGLE scales perfectly with the cluster size. However, we also learned of some query planning issues that still exist in our system with respect to multiple concurrent queries. This challenge is separately addressed in our recent publication [33].

## 8. Conclusions and Future Work

The paper presented our solution, EAGLE, on how to scale the processing pipelines of linked sensor data. The solution includes a system design, a spatio–temporal storage model, a query language proposal, and an extensive set of experiments. The architecture of our design is based on NoSQL technologies, such as OpenTSDB and ElasticSearch, so that we can leverage their scalable indexing and querying components tailored for document, time series, and spatial data. Based on this architecture, we were able to isolate the I/O and processing bottlenecks with the storage model derived from spatio–temporal data patterns. Such patterns are the inputs that drive our data partitioning mechanism for enabling parallel writing and reading behaviors. Therefore, this mechanism makes EAGLE scale better than other state of the art systems as shown in our various experiments. The experiments show insightful quantitative figures on what the scalability issues of other systems and how our solution can overcome such issues. Furthermore, the paper also proposed a query language dedicated for linked sensor data by consolidating recent proposals for enabling spatio–temporal query patterns on SPARQL.

For future work, we intend to integrate a distributed triple store within EAGLE to handle larger non-temporal-spatial data partitions. We are looking into both commercial and open-source clustered RDF stores such as CumulusRDF [45], AllegroGraph [46], Blazegraph (http://www.blazegraph.com/), etc. Furthermore, we are implementing some query optimization algorithms to speed up query performance based on machine learning [33]. Another feature that we want to add in the next version of EAGLE is enabling Allen’s temporal relations by developing additional temporal index algorithms. Finally, to highlight the advantages of EAGLE, further evaluations, such as concurrent read/write loads and detailed system scalibility, will be performed. Furthermore, the comparison of EAGLE’s performance with other well-known distributed triple stores such as GraphDB, Neo4j (https://neo4j.com/), etc., is also needed.

## Figures and Tables

**Table 1:** OpenTSDB row key format.

Element Name	Size
Metric UID	3 bytes
Base-timestamp	4 bytes
Tag names	3 bytes
Tag values	3 bytes
...	...

**Table 2:** Spatial property functions.

Spatial Function	Description
<feature> **geo:sfIntersects** (<geo> | <latMin> <lonMin> <latMax> <lonMax> [ <limit>])	Find features that intersect the provided box, up to the limit.
<feature> **geo:sfDisjoint** (<geo> | <latMin> <lonMin> <latMax> <lonMax> [ <limit>])	Find features that intersect the provided box, up to the limit.
<feature> **geo:sfWithin** (<geo> | <lat> <lon> <radius> [ <units> [ <limit>]])	Find features that are within radius of the distance units, up to the limit.
<feature> **geo:sfContains** <geo> | <latMin> <lonMin> <latMax> <lonMax> [ <limit>])	Find features that contains the provided box, up to the limit.

**Table 3:** Supported units.

URI	Description
units:kilometre or units:kilometer	Kilometres
units:metre or units:meter	Metres
units:mile or units:statuteMile	Miles
units:degree	Degrees
units:radian	Radians

**Table 4:** Temporal property functions.

Temporal Function	Description
?value **temporal:sum** (<startTime> <endTime> [<‘groupin’ down sampling function> <geohash prefix> <observableProperty>])	Calculates the sum of all reading data points from all of the time series or within the time span if down sampling.
?value **temporal:avg** (<startTime> <endTime> [<‘groupin’ down sampling function> <geohash prefix> <observableProperty>])	Calculates the average of all observation values across the time span or across multiple time series
?value **temporal:min** (<startTime> <endTime> [<‘groupin’ down sampling function> <geohash prefix> <observableProperty>])	Returns the smallest observation value from all of the time series or within the time span
?value **temporal:max** (<startTime> <endTime> [<‘groupin’ down sampling function> <geohash prefix> <observableProperty>])	Returns the largest observation value from all of the time series or within a time span
?value **temporal:values** (<startTime> <endTime> [<‘groupin’ down sampling function> <geohash prefix> <observableProperty>])	List all observation values from all of the time series or within the time span

**Table 5:** Dataset.

Sources	Sensing Objects	Historical Data	Archived Window
Meteorological	26,000	3.7 B	since 2008
Flight	317,000	317 M	2014–2015
Ship	20,000	51 M	2015–2016
Twitter	–	5 M	2014–2015

**Table 6:** Categorizing queries based on their complexity.

Category	Non Spatio–Temporal Query	Spatial Query	Temporal Query	Full-Text Search Query	Mixed Query
**Query**	Q2, Q11	Q1	Q5, Q6	Q8, Q9	Q3, Q4, Q7, Q10

## Appendix A. Query Characteristics

**Table A1:** Benchmark query characteristics on linked meteorological data.

Query	Parametric	Spatial Filter	Temporal Filter	Text Search	Group By	Order By	LIMIT	Num. Variables	Num. Triple Patterns
1	✓	✓						3	3
2	✓							3	4
3	✓		✓					7	8
4	✓	✓	✓		✓	✓	✓	7	8
5	✓		✓		✓	✓		4	5
6	✓		✓					5	8
7	✓	✓	✓			✓	✓	7	8
8				✓		✓		3	4
9				✓	✓			6	7
10	✓		✓	✓	✓			7	9
11					✓			2	1
12	✓		✓						
13	✓		✓						
